# The Modes of Evolutionary Emergence of Primal and Late Pandemic Influenza Virus Strains from Viral Reservoir in Animals: An Interdisciplinary Analysis

**DOI:** 10.1155/2011/861792

**Published:** 2011-11-15

**Authors:** Dany Shoham

**Affiliations:** The Begin-Sadat Center for Strategic Studies, Bar-Ilan University, Ramat Gan 52900, Israel

## Abstract

Based on a wealth of recent findings, in conjunction with earliest chronologies pertaining to evolutionary emergences of ancestral RNA viruses, ducks, *Influenzavirus A* (assumingly within ducks), and hominids, as well as to the initial domestication of mallard duck (*Anas platyrhynchos*), jungle fowl (*Gallus gallus*), wild turkey (*Meleagris gallopavo*), wild boar (*Sus scrofa*), and wild horse (*Equus ferus*), presumed genesis modes of primordial pandemic influenza strains have multidisciplinarily been configured. The virological fundamentality of domestication and farming of those various avian and mammalian species has thereby been demonstrated and broadly elucidated, within distinctive coevolutionary paradigms. The mentioned viral genesis modes were then analyzed, compatibly with common denominators and flexibility that mark the geographic profile of the last 18 pandemic strains, which reputedly emerged since 1510, the antigenic profile of the last 10 pandemic strains since 1847, and the genomic profile of the last 5 pandemic strains since 1918, until present. Related ecophylogenetic and biogeographic aspects have been enlightened, alongside with the crucial role of spatial virus gene dissemination by avian hosts. A fairly coherent picture of primary and late evolutionary and genomic courses of pandemic strains has thus been attained, tentatively. Specific patterns underlying complexes prone to generate past and future pandemic strains from viral reservoir in animals are consequentially derived.

## 1. Introduction

The historical emergence and pandemic potency of influenza type A virus—a prominent anthropozoonotic single-stranded segmented RNA virus (family Orthomyxoviridae)—have long constituted challenging phenomena. The Greek physician Hippocrates, the “Father of Medicine,” first described influenza in 412 BC [1]. The name “influenza” was derived from the belief of Italian astrologers in the Middle Ages that the periodic appearance of the disease was in some way related to “influence of heavenly bodies” [2]. Rather earthily, the French named influenza as “the grippe,” suggesting the acute onset of illness, upon which the patient suddenly was seized or gripped by the disease [3]. Yet still recently, influenza has been seriously attributed to introduction of viruses from the space, due to meteorological processes [4]. As far as the origins of life are concerned at large, it has been proposed that cometary ice might have embodied the provenance of earliest precursors of viruses in general on Planet Earth and perhaps cosmically [5].

Influenza pandemics are generated by type A of the causative virus, which is primarily hosted by numerous animal species, chiefly avian, while aquatic birds comprise the principal reservoir. The main historical milestones underlying the course that led to the primal emergence of pandemic influenza strains are for the most part concerned with animal domestication, presumably (Table 1). Lately, the unforeseeable appearance and complicated phylogenesis of the 2009 pandemic swine H1N1 influenza strain, completed (for now) a 91-year period, throughout which five pandemic strains bearing fully analyzed genomes surfaced: H1N1 (in 1918), H2N2 (in 1957), H3N2 (in 1968), H1N1 (in 1977), and again H1N1 (in 2009). Earlier influenza pandemics are not fully confirmable. However, during the previous 70 years (from 1847 to 1917), five pandemic strains emerged, the genomes of which are untraceable, but tentative antigenic subtyping was allowed for by seroarcheological surveillance: H1N1 (in 1847), H1N1 (distinctive variant—in 1857), H2N2 (in 1874), H3N8 (in 1889), and H2N8 (in 1900) (Table 3). Prior to 1847, since 1510, eight influenza pandemics are mentioned in referable scientific literature and were featured by mainly their time of emergence and geographic provenance (Table 3).

Taken together, those 500 years since 1510 are yet but a small portion of the entire natural history of pandemic influenza, which is regarded to exist for about 6000 years [18]. Still, in light of the extraordinary importance of influenza viruses and considering that no additional retrospective virological data can be expected, this 500-year period, with its 18 pandemics, is utilized in the present study as a probe. Full attention is paid to the possible inadequacy of that limited temporal probe. Moreover, this period is unrepresentative of the last 6000 years, in that it is marked by major agroanthropological changes, like industrialized livestock farming, human and animal vaccinations, superfluous human and livestock density, and worldwide transgressing mobility, which certainly affect virus ecology and evolution. This means that any clustering derived out of that 500-year period might ostensibly be unremarkable. However, taking into account the relative genomic stability of the various avian and mammalian—including human—hosts of the virus throughout the recent millennia, it is assumed that, fundamentally, certain cardinal host-virus relationships and resultant interactions have steadily been persisting during that period of time, hence may evenly underlie ancient, recent, and future formations of pandemic strains. The informative period from 1510 to 2010 is therefore reckoned here as a usable probe for the two following purposes.

Featuring virological patterns or common denominators and, conversely, variability or flexibility, underlying the mode of emergence of past pandemic influenza strains, as expectable attributes of future pandemic strains, extrapolatively.

Apply the same featuring for retrospective reconstruction of the primary modes of emergence of primordial pandemic influenza strains, evolutionarily, in that ongoing origination of individual pandemic strains may properly reflect or follow the phylogenesis of primordial pandemic strains, as derived from viral reservoir in animals.

Such perspective would comprise an essential component for an attempt to comprehend and conceptualize the prepandemic and pandemic complexes related to influenza A viruses, from their earliest beginnings onwards. The present analysis explores, then, the appearances of past pandemic strains during the last 500 years, so as to identify both regularity and plasticity that mark the modes of their genesis and emergence. The crucial role of antigenically and genomically shaped virus-host interfaces, alongside with contributive anthropogeographic and zoogeographic elements, is thereby pointed out, multidisciplinarily, within the long-lasting evolutionary derivation of pandemic influenza strains from viral reservoir in animals. 

## 2. Definition and Essence of Pandemic Influenza Strains

The nature of pandemicity is rather not plain. Past influenza pandemics were characterized by a shift in virus antigenic subtype, shift of the highest death rates to younger populations, successive epidemic waves, higher transmissibility than that of seasonal influenza, and differences in impact in various geographic regions [19]. The current H1N1 swine-originated flu virus is the first one categorized as a pandemic strain though not bearing any new antigenic subtype, in comparison with the prevailing strains. This turn depreciates hitherto solid postulations and concepts that a *pandemic strain* is per force afforded by an *antigenic shift*, thereby illustrating certain artificiality, which marks at least one of those two terms, within this context.

The World Health Organization cumulative definition of pandemic influenza—as revised in April 2009—is composed of 6 phases [20], of which the first four represent a potential pandemic strain, in that the latter

- contains at least one animal influenza gene segment;
- has the capacity to infect humans;
- exhibits sustained transmissibility among humans.

The two additional phases represent the transition from potential to actual pandemicity—certainly a meaningful shift, in its essence—but are somewhat artificial, in that they mostly rely on man-defined geographic entities. Even with reference being made to the first four phases, they do not take into account that an entirely human reassortant genome given rise to by the long prevailing human H3N2 and H1N1 strains, instantly—hence not bearing any animal virus gene—could possibly constitute a new pandemic virus, with a new antigenic combination, namely, H3N1 or H1N2. Both biological transmissibility (which is independent of herd immunity) and in effect transmissibility (which is herd immunity dependent) of such reassortant strains—pending, to a considerable extent, on the composition of the internal protein genes, as well—might be effectual. In actuality, an H1N2 strain which emerged during 2001, as an entirely human genetic reassortant between H1N1 and H3N2 subtype viruses spread into many regions of the world and have predominated over H1N1 viruses in several countries [21].

Also, the definition of pandemicity does not consider degree of virulence, extent of cross-antigenicity with prevailing strains, and level of existing herd immunity against a new pandemic strain, although those parameters are essential virologically and in terms of public health. Virulence, for instance, might potentially reach an extreme degree, in the form of cytokine storm, which is often fatal, or cause but marginal mortality—a highly important quantitative parameter—as is the case with the 2009 pandemic strain. A qualitative, genomic parameter, like the presence of animal genes, which the present definition of pandemic strain does include, remarkably materialized within the 2009 pandemic strain, being reassortant of four strains, with five porcine and two avian gene segments. During the spread of the 2009 swine flu virus, it became evident, though, that herd immunity against a given prevailing antigenic subtype cannot hamper proliferation of a new pandemic strain affiliated with the same antigenic subtype, meaning that an antigenic shift is not a prerequisite for pandemicity. Yet, this latter attribute is fully consistent, as well, with the seasonal global spread of new antigenic drift variants—usually called “seasonal strains,” and hitting the very young and elderly—of the given, completely human, prevailing subtypes (presently H1N1 plus H3N2 and previously H2N2). Further, ongoing formation of unproductive, antigenically identical seasonal variants that would manifest themselves pandemically in case there is not any level of already existing herd immunity against them (whether due to natural infection or vaccination) at a given point of time currently takes place, in all likelihood. Such feasibility has been evidenced in actuality [22]. This also means that a new strain defined to be pandemic may basically even have less impact than the impact propelled by a new seasonal variant—either reassortant, recombinant, or mutant—derived directly from merely a prevailing human strain (or strains) and bearing no animal influenza genes, hence regarded as a nonpandemic strain. Such high impact has indeed been demonstrated in actuality, while the 1951 seasonal epidemic strain (a variant of the already prevailing H1N1 subtype) common in England, Wales, and Canada was found to be associated with both higher mortality impact and higher transmissibility than the 1957 and 1968 pandemic strains. Surprisingly, in Liverpool—considered the “epicenter” of the severe 1951 epidemic—the mortality impact and transmissibility even surpassed the notorious 1918 pandemic [23].

Nevertheless, the presence of at least one animal gene segment within a new strain that spreads across human communities—hence potentially pandemic, by definition—certainly has its own immense significance and usually was accompanied, empirically, by a new antigenic subtype, replacing the prevailing one. Such antigenic shifts represented the emergences of the pandemic strains in 1874, 1889, 1900, 1918, 1957, and 1968 (Table 3). This sense-making principle has been depreciated later, though, in that, since 1977, H1N1 and H3N2 have been cocirculating, and in 2009 a new pandemic strain still having the H1N1 antigenic subtype appeared and disseminated globally. For now, it did not—and doubtfully will—replace the H3N2 subtype. The reason might be quite plain, namely, increasing population density of the human host. 

## 3. Presumed Natural History of Pandemic Influenza Strains

### 3.1. Ancestral Phases

The evolutionary pathways of influenza type A viruses in general, and of their pandemic strains in particular, have long been substantially challenging topics. Although a vast variety of avian species and an appreciable range of mammalian species host influenza A viruses, it is fairly obvious that aquatic wild birds, poultry, man, pigs, and horses constitute the most meaningful hosts, biologically, evolutionarily, and epidemiologically. While this virus markedly infects in nature seals and whales as well, its prevalence within wild land-mammals is mostly unknown, except for boars (*Sus scrofa*) (as later elaborated on broadly), plus recent findings in black-lipped pikas (*Ochotona curzoniae*), in China [24], and raccoons (*Procyon lotor*), in the USA [25].

Plenty of data indicate that the primal host-parasite affinity of influenza A viruses formed towards their pristine aquatic avian hosts—wild ducks, foremost—the intestinal epithelium being the provenance and permissive tissue. Many wild duck species, in particular dabbling ducks (*Anas sp.*), evolved—or initially possessed—full tolerance towards influenza viruses and became their ultimate perpetuators, while other wild birds are often clinically affected respiratorily or systemically. Essentially, this virus has been for a prolonged period of time a nondeleterious avian waterborne intestinal pathogen that but occasionally infected airway epithelium. This occasionality turned out, though, to serve, apparently, as a paramount preadaptation, since the airway epithelium was nevertheless the principal target tissue within the evolutionarily next, usually clinically affected hosts of influenza A virus, namely, mammals, including man. Within that context, a broad virological perspective may rather include the chronological milestones depicted in Table 1, such as the domestication of wild boar (*Sus scrofa*), horse (*Equus ferus*), red jungle fowl (*Gallus gallus*), mallard (*Anas platyrhynchos*), turkey (*Meleagris gallopavo*), and quail (*Coturnix coturnix*). 

It has been estimated that the common ancestors of the present sequences of the two surface antigens of avian influenza viruses (AIVs), the hemagglutinin (HA), and neuraminidase (NA), formed within the last 3,000 years [26]. However, the fact that for many millions of years RNA viruses (in general) and the primordial influenza virus reservoir (wild ducks) coexisted in nature implies that the initial prototypic AIV probably formed much earlier than 3000 years ago; let alone that pandemic influenza viruses—which certainly emerged later than AIVs—are regarded to have arisen about 6000 years ago (Table 1). 

### 3.2. Primary Developments

At any rate, the primary extended adaptation of AIVs to nonhuman mammals preceded adaptation to man, in all likelihood. Wild waterfowl-frequented lake water regularly contains AIVs. The virus is relatively stable in water and can remain viable for up to 200 days, depending on temperature and other environmental factors [27]. Thus, such bodies of water and adjacent shorelines are prone to become contaminated, increasing the chance of subsequent exposure of mammalian species to AIV, too. Assumingly, the very first terrestrial mammals that contracted influenza viruses could be wild boars or wild horses drinking contaminated water, whereby contact between virus and the nasal or pharyngeal epithelium—the target host tissue—occurred. Alternatively, air exhaled by nearby respiratorily infected waterfowl could as well be a source for such contact, which might have propelled the very initial generation of mammalian influenza viruses at large. The latter mode of infection could have been more feasible, in that it involved innately avian viruses that already shaped, basically, as airborne ones, rather than waterborne. An appropriately demonstrative event of productive avian-to-equine transfection scenario took place in 1989 in China, when a genuine avian H3N8 virus transmitted directly from birds to horses, giving rise to a new H3N8 equine virus—A/Equine/Jilin/1/89 (H3N8) [28]. Moreover, occasionally, equine H3N8 viruses are contracted by pigs [29]. Notably, the antigenic subtype H3N8 (out of 144 possible HA-NA antigenic subtype combinations) has been found to be responsible for over one quarter of the influenza infections in wild ducks [30]. 

While the first equine-adapted strains emerged sometime in the far past, after the divergence of a variety of avian HA subtypes, and prior to the appearance of the first human-adapted influenza A strains [31], it has been indicated that porcine-adapted strains surfaced for the first time only toward (namely, in 1917, when outbreaks of influenza in swine occurred), or in 1918, concomitantly with the human Spanish (swine) flu pandemic strain [8]. Presumably, though, swine influenza viruses could have been established within domestic hogs much earlier, in two fashions:

- through the domestication of originally infected wild boars;
- through infection of domestic pigs by AIVs during their millennial coexistence, considering that in China boars were initially domesticated at about 8000 BC, cofarmed with chickens since 6000 BC and with ducks since 4000 BC (Table 1).

The absence of influenza-like illness among swine infectious diseases clinically described before the 1918 swine flu pandemic is probably but ostensible, and even if porcine influenza viruses did not indeed exist in America before 1918 [32], their natural history in the Old World—particularly in Southeast Asia, with its distinctive agricultural features (detailed below)—was conceivably much dissimilar, considering that both wild and domestic hogs were not at all found in the New World until 1539. Illustratively, in that connection, natural introduction of avian H1N1 into domestic pigs in China has been observed, followed by ongoing virus circulation within pigs [33]. Also, an avian H1N1 virus likewise established itself in pigs in Europe [34]. Further, natural infections of swine by avian strains of the subtypes H3N2 and H3N3, H4N6, H5N1 and H9N2 were evidenced worldwide [35]. An avian-originated H2N3 strain, too, naturally formed within pigs [36]. It appears as if such occurrences properly exemplify a direct avian-porcine interface that could have well been evolving and lasting for eras, already.

Equivalent moves presumably underlay the generation of the earliest prototypic porcine influenza viruses in the far past, already involving, then, wild boars—a widespread species (*Sus scrofa*) in the Old World, the same as the domestic pig species—in conjunction with infected wild waterfowl. This possibility is supported, retrospectively, by recent swine influenza virus serological data, available from European wild boar populations. Antibodies to three porcine influenza subtypes, namely, H1N1, H3N2, and H1N2, have been detected in European wild boar populations, in variable concentrations [37]. Seroprevalence may vary from 0% to as much as 75%, depending on country or region and swine influenza virus subtype [38]. The H1N1 subtype seems to be the most prevalent one among wild boars [39]. H3N2 influenza viruses were yet isolated too from naturally infected wild boars in Germany [40]. The sources of that wild boar infectedness could be pigs, humans or birds.

Waterborne and airborne transmission of AIVs to wild boars—known for their preference for wetland and mud—is feasible, as mentioned; besides, wild boars (as well as additional wild omnivorous and carnivorous mammalian species) that are likely to meet wild waterfowl, such as ducks and geese, could contract AIVs from infected healthy, diseased, or dead waterfowl, which they occasionally feed on. A recent study of such 60 wild species revealed, further to swine, that canines—including Chinese wolf (*Canis lupus chanco*), arctic fox (*Alopex lagopus*), and corsac fox (*Vulpes corsac*)—as well as Persian leopard (*Panthera pardus ciscaucasica*), North American striped skunk (*Mephitis mephitis*), and opossums, have AIV receptors [41]. Also, raccoon [25] and pika (a herbivore) [24] were found to regularly contract AIVs, in the wild. These findings imply that the present and historic circulation and evolution of influenza A viruses in the wild are perhaps considerably wider than current common knowledge. Moreover, the established contraction of AIV by a herbivore due to environmental pollution caused by waterfowl—as observed in the study about pika—indicates a potentially paramount mode of transmission to herbivorous species in general, as was apparently the case of the earliest contraction of AIV by wild horses. Being omnivores, wild boars can contract AIV either likewise or though eating infected sick or dead birds, as well. Evolutionarily, in that connection, it might be advantageous for nonvirulent viral strains to occasionally become virulent and cause disease, thereby gaining access to new potential host species inclined to feed on the diseased or dead hosts. Such mechanism has been evidenced in zoo leopards (*Panthera pardus*) and tigers (*Panthera tigris*) that fed on H5N1-infected poultry carcasses [42]. Naturally infected domestic cats (*Felis catus*), as well as the carnivorous mammal stone marten (*Martes foina*), contracted the virus in the wild, presumingly upon feeding on infected birds, too [43]. All in all, the possibility that wild boars could have picked avian influenza strains in the Eastern Hemisphere in the far past, already—much before the ostensibly earliest emergence of porcine influenza strains of 1918—thereby becoming one of the initial mammalian hosts of influenza viruses at large, is indeed plausible. Alternatively, and still much before 1918, domesticated boars could readily contract influenza A viruses for the very first time while cofarmed with chickens and ducks in old China and subsequently persistently circulate them.

At any rate, domestic pigs and horses are regarded as the principal terrestrial mammalian hosts of influenza A viruses, in addition to man. Viral adaptation to mammals was and still is, nonetheless, secondary. It sufficed, however, to shape a widespread, though antigenically quite limited variety of swine-, equine-, and human-adapted influenza A strains that retain—unlike numerous benign enterotropic avian strains—their respiratory-associated pathogenic capacities, foremost. 

### 3.3. Generation of Pandemic Strains

In its fundamental course, the generation of a pandemic influenza strain may progress through three successive stages, each accounting for acquiring:

- infectivity to man (acquired while virus is circulating within animal hosts);
- virulence—rather than subclinical infection—to man, (acquired while virus is circulating within animal hosts or humans);
- airborne transmissibility among humans (acquired while virus is circulating within animal hosts or humans) [44].

Basically, stages (A), (B), and (C) may take place within a wide variety of avian species and certain mammalian species, as well, but in practice, pigs and avian hosts—wild waterfowl and poultry foremost—are more likely to serve for the materialization of those stages. Any of the three stages may evolve by means of various types of genetic changes: mutations, recombinations, and, typical of influenza virus, genetic reassortment with a different collocated virus. Notably, stage (A) necessarily occurs within an animal host and resultant infection of humans is barely traceable, as long as not clinically manifested. Such phenomenon—asymptomatic infections in man—has been observed with respect to influenza [45], and may reflect initial adaptation of a given porcine or avian strain to human host. Human asymptomatic infections have thus been evidenced even with respect to the typically highly virulent H5N1 virus [46, 47]. Such infections may take place due to absence of cytopathic effect, or considerable restriction of the primary infection, and are detectable serologically, through virus isolation, or otherwise [48]. Thereafter, the infective virus may or may not undergo stage (B), and then stage (C), while the order of probabilities, allover, may descend in the following manner:

stage (A) (alone, without there being any succession);
(A) and then (B) (without succession by (C));
(A) and (B) at once (while still infecting the animal host, without succession);
(A), (B), and (C) (successively);
(A) and (B) at once (while in the animal host), followed by (C) (while in the human host);
(A) followed by (B) and (C) at once (whether within the animal or human host);
(A), (B), and (C) at once (while still infecting the animal host) [44].

Although largely theoretical for now, this paradigm covers the entire animal-human interface presumably underlying the formation of a pandemic strain. It illustrates the essentiality and machinery of either gathering or abruptly forming critical mass, ultimately allowing for the realization of stage (C). Relying on interfaces chiefly involving poultry, pigs, and humans, this paradigm is nonetheless being permanently nourished by and dependent on viral genetic dynamics within wild birds. Therefore, the bioanthropological factor of host population density—referring mainly to migratory waterfowl, poultry, pigs, and humans—even if seemingly trivial, may constitute, historically (as well as presently), a critical mass; it would expectedly amplify the overall genetic core and transmissibility rate of a candidate prepandemic strain, both qualitatively and quantitatively, to a tipping point.

Airborne transmissibility of influenza virus is a cardinal prerequisite for persistent epidemic dissemination and pandemicity. Direct or indirect human-to-human nonrespiratory contagion—albeit of considerable importance, and at times successive—would not at all equal ongoing airborne transmission in generating pandemic spread, in the case of influenza [49]. As a matter of fact, any non-aerogenic influenza transmission among humans is by far less efficient, hence may scarcely be considered as propelling a pandemic [50]. It follows that even if one human-to-human airborne transmission occurs, bringing about a productive infection within the contractor, it would suffice [51]. Such is the case, because this would mean that the virus has biologically acquired the ability to proliferate in a fashion leading to local formation (in the host), consequent respirational release (by the host), and subsequent viable dispersal (in the air) of sufficient amount of aerogenically infective virus; therefore, the infection of the contractor as well is prone to likewise yield further effectual airborne transmission, and so on. By contrast, contact transmission alone cannot support pandemicity. This distinction is as valid with respect to current and primordial emergences of pandemic influenza strains.

The viral interface of influenza between pigs and man is apparently by far more intense and productive than between any other animal and man [52]. This interface is also the oldest one, in terms of domestication, because hogs may well represent the very first domesticated wild host that was regularly infected by influenza A virus. Moreover, the earliest domestication of wild boars took place in China about 4000 years before the initial emergence of pandemic strains and roughly in parallel with the earliest domestication of wild chicken (red junglefowl—*Gallus gallus*—a species not regularly infected by, though sensitive to, influenza, in nature), in nearby Vietnam. Further, domestic chicken was introduced into China 2000 years before the initial emergence of pandemic strains, and earliest domestication of wild ducks (mallards—a species regularly infected by influenza in nature) took place, in China, 1000 years before the initial emergence of pandemic strains (Table 1). This chronology may imply that, during those 1000–2000 years, the primary creation of the triangular genetic melting pot that includes humans, pigs, and birds—basically the pristine melting pot giving rise to at least the past 1918 and the recent, 2009 pandemic swine flu strains—has been shaping. Virologically, it means that some certain conjunction of porcine and avian influenza virus genes possibly yielded, then, a reassortant virus that for the very first time infected man productively and at the same time—or at some later point of time during those 2000 years—attained complete self-sustained transmissibility within human populations, namely, pandemicity, as well. A crucial transition to a fully airborne, respiratorily communicable pathogen thereby occurred during that course. Initial infectivity towards man could have been afforded by swine strains, and—deducing from the necessity of avian genes for pandemicity, as empirically observed with regard to the genomes of the pandemic strains since 1918 onwards—initial pandemicity could have been afforded by means of avian genes primarily donated by ducks. Concomitant contribution by chickens could have taken place mainly in terms of amplification, presumably. All in all, it appears that the first one of the following four possibilities is most likely:

- the genesis of primordial pandemic strains was propelled in China by the conjunction of domesticated pigs (as essential factor) and domesticated birds, ducks in particular (as crucial factor); or
- by domesticated birds merely,
- by domesticated pigs merely,
- by some other cause.

The avian-porcine-human conjunction has likely since been a long-lasting shaper of pandemic strains, in China and beyond, although the sheer avian-human conjunction was as well productive in that concern, contemporarily. The initial domestication of wild horses in nearby Kazakhstan apparently had but a limited—yet still significant—input in that sense, and the initial domestication of wild turkeys in Mexico has probably been appreciably contributive, particularly consequent to the primary introduction of domestic pigs from Europe to Mexico and ongoing cofarming. Within the enormous variety of avian species that host influenza viruses, only turkeys, quails, and wild ducks are naturally infected by porcine strains (as later elaborated on), hence are meaningfully more involved in the human-porcine-avian genetic melting pot. At any rate, while donation of avian virus genes is in all likelihood vital for the formation of any pandemic genotype (as evidenced below), intact avian strains can productively infect humans, but are not aerogenically transmissible among humans, hence incapable of generating a pandemic (as is predominantly the case with the highly pathogenic avian H5N1 virus, for now). Intact avian strains can reassort with human strains, though, and thereby form pandemic strains.

## 4. The Fundamental Significance of Viral Involvement into Animal Domestication

### 4.1. Domestication of Birds

Birds (Class Aves) consist in 27 orders, taxonomically. It so happened that three orders in particular (among the enormous variety of avian species that host influenza A viruses) constitute the most important ones as influenza A virus hosts, while members of two of those three orders—waterfowl (Anseriformes) and landfowl (Galliformes)—were domesticated thousands of years ago, and chiefly comprise poultry farming until present. Wild waterfowls are natural reservoirs of influenza type A, whereas landfowls are apparently insignificant in the wild, in that regard, but are frequently infected as poultry. The third order (Charadriiformes) includes gulls, terns, and shorebirds, which markedly contribute to the circulation and diversity of AIVs in the wild. Gulls are known, as well, for their close affinity to human settlements. Waterfowl and landfowl are evolutionarily and phylogenetically closely related [53] hence their domestication and co-farming could have readily facilitate the initial transfection of domestic land-fowl by domestic waterfowl-harbored influenza strains. Connectedly, the remarkable tolerance of ducks towards influenza viruses, as opposed to the extreme clinical susceptibility of chickens, recently led to the identification of a duck gene called RIG-I, which carries the code for a protein that immediately detects the RNA of influenza virus after the virus invades the duck's lung and tracheal cells. It then sets off a chain reaction inside those cells to help fight off the disease. This crucial gene is absent within chickens, a finding that offers, potentially, the creation of a resistant—yet tolerant—transgenic chicken breed [54]. In such case, the main sector of poultry worldwide—chickens—would gain a cardinal advantage, but this might amplify the genetic melting pot responsible for the genesis of pandemic strains.

On the whole, mankind has changed the natural ecosystems of birds through captivity, domestication, agriculture, and commerce, which began thousands of years ago, and continues through today [55]. This has profoundly transformed the existence of low pathogenic avian influenza (LPAI) viruses from being a diverse group of viruses circulating asymptomatically in certain free-living aquatic birds to also becoming a less diverse group of influenza A viruses, causing endemic respiratory disease in horses, pigs, man, and domestic poultry. The concerned man-made systems are very heterogeneous and include hobby, village, and rural poultry; fighting cocks; captive wild birds; outdoor-reared noncommercial and commercial poultry; industrial indoor-reared poultry. Significant differences between the Old World and the New World are noticeable, though, which have important virological, evolutionary, and epidemiological implications on pandemic strains.

### 4.2. China and Southeast Asia

The first domesticated bird worldwide—taking shape, then, into chicken—was the red jungle fowl, which is uniquely native to and a common species in Southeast Asia [56], but it is not known whether it plays any role in the circulation of influenza viruses in the wild. It is therefore unclear whether this species could have been already infected prior to its domestication. By contrast, it is likely that some of the first domesticated mallards—in China—could have originally been influenza infected, subclinically, and if not, the presently prevalent fashion of contracting the virus by domestic ducks—meaning from collocated infected wild ducks—could have readily been the mode of their very initial infection, in the far past. Domesticated mallards became, in that case, the ever first avian influenza reservoir adjacent to humans, in China. This reservoir might have served as a source for initial and recurrent transfections of humans, domesticated landfowl, and additional domestic waterfowl, as is the case of today's influenza A virus dynamics. Likewise, there is seemingly no reason to think that the currently productive infectious interface between poultry and domestic pigs could not have been formed consequent to their established co-farming in China, already thousands of years ago, especially subsequent to the domestication of mallards in China.

This fundamental, anthropologically derived ecosystem has been expended in Asia by the domestication of horse, goose, quail, pheasant, and guinea fowl, all contributing, presumably, to increasing prevalence and variety of the virus, hence indirectly to the chances of pandemic strain formation. For example, pheasants—an unnoticed domestic host which is natively widespread in Eastern and Central Asia—were shown to support prolonged subclinical influenza infection and shed virus for many weeks [57]. Moreover, quails—originally domesticated and presently widespread in Southeast Asia—were found to regularly maintain influenza H9N2 strains that are incorporated into human virus inventory [58]. In that connection, most avian and human influenza viruses preferentially bind to specific epithelial receptor types having SAR2,3Gal (avian receptor)- or SAR2,6Gal (mammalian receptor)-terminated saccharides, respectively [59]. Both receptors were typically identified in the respiratory tract of swine [60], providing solid molecular evidence for pigs as major “mixing vessels” for human, and avian influenza genomes, of which pandemic strains can be derived. The role of swine in that regard is pivotal. Importantly, yet, these receptor types are also found in the respiratory tract of man [61] and quail [62]. In the human respiratory tract, avian receptors are concentrated, though, in and around the alveoli, rather than the upper airways, thereby hampering airborne transmission of innate avian influenza viruses among humans [63]. Recently, comparative quantitative findings were obtained regarding the presence of human and avian receptors in chicken, duck, and turkey [64]. The main findings refer to both tracheal and intestinal epithelium and illustrate considerable essentiality of the former, whereas the latter is significant in relation to tentative contraction of human strains by chicken only (Table 2).

Those findings are in conformity with experimental infections of chickens and ducks. Infectivity of innately human epidemic strains to chickens has indeed been demonstrated, though but experimentally, using human H3N2 strains [65, 66]. Similarly, human H1N1 and H3N2, as well as swine H1N1, experimentally showed infectivity to ducks, replicating, though, in the upper respiratory tract only [67]. Another human epidemic H3N2 strain—with no infectivity towards ducks—was used as a donor of various genes, in conjunction with a duck-originated virus (H2N2), to induce reassortant viruses that were employed for experimental infection of ducks [68]. It was thus found that only reassortants possessing human M or NS gene and the remainder from the avian virus replicated in duck intestine. Notably, while farming of chickens and ducks is common in Southeast Asia, turkeys are very rare across this region, but are common—alongside with chickens—in America. Apparently, this dissimilarity influenced the variant modes of genesis of pandemic strains, as shown below. In Southeast Asia, nevertheless, domestic ducks uniquely have close interface with feral and wild ducks, an interface which immensely contributes to the genetic dynamics of both avian and mammalian influenza viruses.

### 4.3. Mexico and the Rest of America

In America, as compared with Asia, a different course took place, leading to co-farming of domestic land-fowl and pigs. In that case, a peculiar biotic conjunction—not following any natural ecosystem, and yet appreciably affecting the molecular epidemiology of influenza—formed through the initial domestication of wild turkeys in Mexico, and the earliest introduction of domestic pigs into America—specifically the Gulf of Mexico coasts—later on. Wild turkeys are found only in America. The Mexican wild turkey (*Meleagris gallopavo gallopavo*), which is the nominate subspecies, was initially domesticated by the Aztecs, giving rise to the worldwide domestic turkey. Descendants of the original Mexican domesticated turkeys were later on introduced into Europe by the Spanish [69].

Conversely, domestic hogs were initially introduced from Europe into America, a continent which has innately been deprived of wild boars. In 1539, a Spanish vessel carrying for the first time European domestic hogs destined for the Gulf Coast [70]. Intentional or accidental release of animals derived from these stocks likely represents the source of the first feral pig populations in Mexico plus the Gulf and southeast regions, as well as the continental USA. Feral pigs currently found in the USA constitute a combination of descendant lines of European wild boars originally released for sport hunting purposes, and feral animals derived from escaped domestic pigs; these readily interbreed where they co-occur [71].

Feral swine populations in America are rapidly expanding in both numbers and range and are increasingly coming into contact with waterfowl, humans, and agricultural operations [72]. Remarkably, the highest seroprevalence to swine influenza viruses among feral swine in the USA—14.4%—was found near Mexico, in Tex, USA. All seropositive swine were exposed to H3N2 subtype, while in San Saba County, Tex, USA, of the 15 seropositive samples, 4 were positive for H1N1 and 7 for both H1N1 and H3N2. Notably, there was large geographic and temporal variation in antibody prevalence but no obvious connection to domestic swine operations. From these results, it is apparent that influenza in feral swine poses a risk primarily to swine production operations and hence to humans as well, secondarily. However, because feral swine share habitat with waterfowl, prey on and scavenge dead and dying birds, are highly mobile, and are increasingly coming into contact with humans, the potential for these animals to become infected with avian and human influenza in addition to swine influenza is regarded a distinct possibility [72]. Referring—conceivably at least—to this formation of influenza infected feral swine populations as a reversible process, it would in parallel represent, in that sense, the converse feasibility that wild boars domesticated for the first time in history, in China, were originally influenza-infected, to an appreciable extent. In America, co-farming of pigs and turkeys has been taking place since the 16th century to the present. Assumingly, during those 500 years, bidirectional viral interface evolved, uniquely, between pigs and turkeys. When domestic pigs were introduced into America from Europe, chicken farming already existed in America (following initial introduction from Polynesia) [73], but turkeys constituted the predominant domestic land-fowl. The bi-directional viral interface thus shaped in America, primarily between pigs and turkeys, was probably propelled by porcine—rather than turkey—influenza strains, in light of the fact that none of 210 sampled wild turkeys had antibodies to influenza, indicating that populations of native wild turkeys are rather unimportant in the epidemiology of this virus [74], and, therefore, that domesticated turkeys apparently were not infected prior to their domestication. However, domestic pigs initially introduced to America from Europe could be originally infected or thereafter contract the virus from humans or waterfowl.

Noticeably, until 1998, only classical-swine H1N1 viruses were isolated from the USA domestic pig population [75]. For nearly 70 years, this H1N1 lineage of North America has been relatively stable as the predominant and only porcine antigenic subtype [76]. This lasting stability may reflect local uniqueness which possibly poses America, historically, as a principal melting pot of pandemic swine-derived H1N1 strains at large, worldwide, considering that the last three known pandemic H1N1 strains (regardless of the controversial 1977 H1N1 strain) emerged in America, of which two were porcine originated (1918 and 2009) and one unidentifiable (1857) (Table 3). Pig husbandry is very common over most parts of the Eastern Hemisphere too, hence this uniqueness of America, if true, has to have stemmed from certain local distinctiveness, which is designed, seemingly, by the relative exclusiveness of the North American avian lineage of influenza viruses circulating in both wild birds and poultry [77]. Besides, domestic turkeys co-farmed with pigs in America (USA), uniquely, constitute the only avian species known to ordinarily contract porcine—and, at large, mammalian—influenza strains, (H1N1, H3N2 and H1N2), worldwide [78]. 

Also unique to America and turkeys, as far as known, is the noninduced contraction of intact human strains by an avian species. Direct transmission of the 2009 H1N1 pandemic strain from human to turkey thus occurred, occasionally, in America (Chile, USA, and Canada), owing to artificial insemination of turkeys, and generating local outbreaks in turkey flocks; when experimentally emulated, infection occurred and was followed, significantly, by respiratory virus secretion [79]. Such human intervention, albeit unintentional, might have deleterious corollaries in terms of recirculation of viral genomes with pandemic potential within avian populations. Moreover, actually, those three human-to-turkey swine-originated influenza transfection episodes were detected because they caused outbreaks in turkeys, hence there might be additional ones that were not detected, due to possible asymptomatic infection.

Furthermore, globally, the only three documented emergences of swine influenza strains infecting humans in an epidemic (the New-Jersey strain in 1976) or pandemic (in 1918 and 2009) fashion took place in North (including Mexico) America (all those strains were of the H1N1 subtype). Even once, this never occurred in Asia or elsewhere, as far as known. While pigs and chickens are abundant in both North America and Asia, there is a major difference in that turkeys are prevalently farmed conjunctively in North America, whereas ducks constitute the analogous co-farmed poultry in Southeast Asia. This is a distinctive agroanthropological feature, with far-reaching virological implications. This vicariousness has been accentuated, too, in that an American swine strain of the subtype H3N2 (triple reassortant including human genes) was experimentally shown to transmit efficiently both ways between swine and turkeys, replicate and transmit among turkeys, replicate without transmission among chickens, and be noninfective towards ducks [80].

The origin of domestic turkeys is North (including central) America, and their domestication took place in Mexico. Pigs are the only species known to regularly and commonly contract human strains worldwide, and turkeys are the only species (regardless of man) known to ordinarily contract porcine strains. The latter frequently contain genes of human strains. Pigs regularly contract viruses from turkeys (and other birds), often account for mammalian-avian genetic reassortment events, and transmit various reassortant viruses to humans. Altogether, this means that there might be some unique contribution afforded by turkeys (typically in America)—but not by ducks (in Asia)—to porcine-harbored influenza genomes, which infect turkeys and later on infect again pigs, thereby attaining, possibly, infectivity towards and transmissibility among humans. Such uniqueness could underlie the globally singular three emergences of contagious swine flu in humans in North America (two pandemics; one epidemic, in 1976), as mentioned above and elaborated on below.

Basically, the contributive potential of turkeys to the emergence of pandemic strains has indeed been pointed at [81], in light of the infectedness of turkeys with swine influenza viruses in North America. Significantly, the anthropologically formed conjunction—in 1539—of domestic pigs (Western Hemisphere originated) and domestic turkeys (Eastern Hemisphere originated) can be biomedically regarded, in that sense, as a fundamental unbalancing human intervention. Alongside, an influenza-resembling epidemic that broke out in 1493 in the Antilles, after the arrival of Christopher Columbus, killing in less than a quarter of a century almost the entire indigenous population, was the first epidemic of human influenza in America, reportedly [82]. Avian influenza viruses most probably prevailed much earlier in America, at any rate. Biogeographically, it is notable that wild mallards—possibly the most important host of influenza A viruses across the globe, both historically and presently—which are today widespread in North America, apparently mostly derived in that sub-continent from some males that arrived from Siberia in the far past, settled down, and mated with American black duck (*Anas rubripes*) ancestors [83].

## 5. Referable Influenza Pandemics during the Last 500 Years

### 5.1. Profiling the Last 18 Pandemic Events

Given unavoidable scientific difficulties in applying the definition and essence of influenza pandemicity with respect to seeming influenza pandemics that preceded the 1918 pandemic, a bibliographically multisourced list of putative influenza pandemics that took place since 1510 has been comprehensively annotated in the present analysis and is presented in Table 3. Pandemics that occurred before the year 1510 are largely untraceable, although that year does not substantially mark anything, anthropologically or virologically. All over, the list contains 18 pandemics, which reputedly took place from 1510 to 2010. Basically, those 18 pandemics are traceable in terms of time and place; out of them, the last 10 pandemics strains are regarded as antigenically identifiable and the very last 5 ones are genomically recognized.

#### 5.1.1. Remarks

Due to the complexity of annotating Table 3, the following remarks should be made regarding the pandemics that took place since 1989, as mentioned in that table:

1989—equine resembling strain; cocirculation with the preceding H2N2 strain,
1900—apparently an interhuman reassortant of the two preceding strains,
1918—Porcine resembling strain,
1957—reassortant of the preceding strain; reassortment host unknown,
1968—reassortant of the preceding strain; reassortment host unknown,
1977—resurrected reassortant strain; cocirculation with H3N2 strain,
2009—porcine originated strain; cocirculation with the two preceding strains.

#### 5.1.2. Geographic Provenance

The Americas, at large, may pertain to North and South America; yet considering Panama, Mexico, and the USA as the mere recognized pandemic provenances in the Americas, the concerned sub-continent (namely, North America) may likely originated the 1761 pandemic, as well.

Russia, at large, pertains to both Asia and Europe, although Asiatic Russia seems to basically be more plausible as pandemic provenance, either Siberia or the formerly Soviet southern republics (as was the case of the 1889 pandemic, then designated “Old Russian Flu,” while actually originated in Kazakhstan and Kyrgyzstan).

#### 5.1.3. Antigenic Subtypes

Although the antigenic subtypes prevailing during the period 1510–1830 are untraceable, there are clear indications regarding their recycling throughout those 320 years [85]. Some or all of those subtypes were probably identical to the recognized HA and NA subtypes that appeared later on, during 1847–2009, but other subtypes cannot be excluded.

The remarkable phenomenon of recycling pandemic influenza antigenic subtypes has been thoroughly revisited, with reference being made to the 19th century; it was thereby pointed out that the 1889 pandemic caused by the subtype H3N8 had been preceded by the subtype H2N2 [87]. The findings presented in the latter paper rather imply, as well, that the two subtypes have apparently been cocirculating, from 1889 until 1900.

According to Taubenberger and Morens [85], the antigenic subtype that prevailed from 1847 to 1855 had protective effect against the H1N1 1918 swine flu, while the virus that appeared in 1857 did not have, although regarded to represent another (or an additional) wave of the same epidemic/pandemic (and same antigenic subtype). 

### 5.2. Interpretable Pandemic Strains

#### 5.2.1. The African-Originated 1510 Pandemic Strain

This was the first referable influenza pandemic globally and the only one originating from Africa [85]. In Europe, it then initially appeared in Sicily [3], implying, apparently, of a coastal North African provenance, namely, within the range of the Northern Hemisphere. However, although clearly being an exception (as the only case of an African provenance), it may nevertheless indicate that, basically, pandemic strains could emerge anywhere in the Northern Hemisphere (if not in the world). The huge, densely populated agricultural area of the Egyptian Nile Delta—an intercontinental axis of numerous migratory birds, as well as the source of the remarkable avian influenza epidemic of 1945 [91], plus the unique avian-derived human strain H10N7, and presently a solid, solely lasting endemic focus of the virulent semianthropophilic H5N1 virus in Africa—could have possibly support, then, the emergence of the 1510 pandemic strain.

#### 5.2.2. The 1889 H3N8 Equine-Resembling Pandemic Strain

The antigenic combination of H3 together with N8 typically represents one of the two mere subtypes of equine influenza (namely, H3N8 and H7N7), known for a long period of time to clinically and epidemically infect horses. The 1889 pandemic was reportedly caused by the subtype H3N8 (Table 3). Geographically, it appears that the most thorough study done about this pandemic indicates its origination in Kazakhstan and Kyrgyzstan [88]—basically parts of Asiatic Russia. Those countries are well known for their remarkably developed horse husbandry, particularly during the 19th century, and Kazakhstan is the land where wild horses were primarily domesticated, worldwide. Notably, the subtype H3N8 is rarely found within pig populations, but is prevalent, subclinically, within avian hosts. It is hence reasonable to suppose that the 1889 H3N8 pandemic was generated by an equine virus, or, perhaps more likely, by a reassortant virus bearing equine genes together with human and/or avian genes. This scenario seems to have been somewhat more probable than the converse one—namely, that an H3N8 virus initially infected humans, thereafter transmitting to horses—although the latter scenario is not unfeasible. Eighty years later, noticeably, possible phylogenetic involvement of an equine H3N8 virus preceded the contribution of the HA and PB1 genes by an avian strain to the formation of the pandemic 1968 H3N2 strain [92]. Interestingly, the innately equine H3N8 strain A/equine-2/Miami/63 was found to be experimentally infective towards humans, and virus shedding ensued in all subjects. The most common clinical response was a febrile illness indistinguishable from naturally occurring human influenza [93]. Also, it has been recently accentuated that historical, observational, and experimental data suggest that equine influenza viruses may also infect man and thus have the potential for generation of pandemic viruses [94].

Different from the data presented in Table 3 and the related remarks (already mentioned and to be elaborated on), there are sources noting that a pandemic H3N8 subtype prevailed in 1874, H2N2 in 1890, and H3N2 in 1902. If this is indeed the case, it is plausible that the 1874 H3N8 pandemic strain—the geographic provenance of which is not indicated—originated in North America from the potent—supposedly H3N8—equine epizootic virus that catastrophically afflicted this subcontinent in 1872-1873. That outstanding epizootic became known as the “most destructive recorded episode of equine influenza in history” [95]. Contact between humans and infected horses was then unpreventably very common, in all likelihood, and could have possibly brought about human infections that gave rise to a pandemic strain.

#### 5.2.3. The 1900 H2N8 Pandemic Strain

This pandemic was generated by the last unrecoverable pandemic strain, yet a partial profile of this strain can be figured out. In principle, it could have been derived as a reassortant virus of the two presumably cocirculating strains H2N2 and H3N8 that preceded it (Table 3). Such course may be regarded as a feasible one, and assuming that it did take place, then it was the only case during the 500 years probed in this study, whereby an intrahuman reassortant emerged as a pandemic strain. Further, it has been indicated that the 1918 H1N1 pandemic strain originated (in pigs) as a reassortant virus and that part of its genes were derived from the 1900 pandemic strain [89].

## 6. The Potency of Porcine H1N1 Strains

Swine influenza is known to be caused by influenza A subtypes H1N1, H1N2, H2N3, and H3N2 [96]. Occasionally, pigs contract AIVs, like H5N1, H7N7, or H9N2, and the equine H3N8 virus [29], too. Three influenza A virus subtypes—H1N1, H1N2, and H3N2 (of which H1N1 and H3N2 are equivalents to pandemic strains, antigenically)—are the most common strains within pigs worldwide, while the subtype H1N1 is the most significant one. Curiously, the sole mammalian isolates bearing the related antigenic combination H1N3 were but once obtained from a whale and a human being, [97] the related combination H2N2 has been isolated, among mammals, only from man, causing a pandemic in 1957, and the last related combination, H2N1, has never been isolated from any mammalian species. Occasionally, humans are transfected by porcine strains, but usually those strains are nontransmissible thereafter within humans. Certain porcine-originated H1N1 strains are yet of particular importance, as follows.

### 6.1. 1918 H1N1—Spanish Flu Pandemic

This outstanding pandemic, named also the 1918 swine flu pandemic because of prevalent contemporary influenza morbidity within pigs, has been infamous in that it killed, roughly, 60–100 million people worldwide. The causative strain emerged in the USA, probably in pigs, initially [89]. What was referred to as the “Spanish flu” seems to have originated, though, in North America. Belligerents on both sides of the Atlantic did not make public details of the flu outbreak because of the effect on troop strength, which had important battlefield implications. Spain was a noncombatant in World War I and was the only country to make public details of their influenza experience in 1918 [3]. Historian Alfred W. Crosby observed that the pandemic originated in Kansas and was echoed with reference being made to Haskell County, Kansas, as the likely point of origin. In terms of recorded human cases worldwide, the disease was first observed among soldiers at Fort Riley, Kansas, on March 4, 1918 [98]. Also, it has been contended that a precursor virus was likely to have first come from China, mutated in the USA, and then broke out [99].

Thus far, only the 1918 pandemic strain has paleoanthropologically been recovered, and the following case is notable, in that respect. On the Seward Peninsula of Alaska, Brevig Mission (called Teller Mission in 1918) suffered extremely high mortality during the influenza pandemic in November 1918. Individual records were not available, but historical records show that influenza spread through the village in about 5 days, killing 72 people, representing about 85% of the adult population. Victims were buried in a mass grave in permafrost. In August 1997, four of these victims were exhumed. Frozen lung tissues were biopsied in situ from each. Although the histological analysis was hampered by artifacts of freezing, these tissues showed evidence of acute massive pulmonary hemorrhage and edema. One of the victims, an Inuit female, was influenza RNA positive A/Brevig Mission/1/18 (H1N1) [100]. No viable virus could be isolated from the specimens, but the pathogen was recreated on the basis of its deciphered genomic sequence. The resurrected strain has subsequently been utilized for experimental infections, through which its salient virulence and lethality were found to be the outcome of its ability to induce an overreaction of the immune system in the form of cytokine storm [101], as well as to invade the lungs and cause aggressive pneumonia [102].

The genomic origin of this pandemic strain is not fully traceable. The causative agent was claimed to bear an entirely avian genotype [103], and later on pointed at to be a triple reassortant involving porcine, avian, and human strains [104, 105]. Also, it has been observed that the HA gene of this virus was a corollary of genetic recombination [106]. Remarkably, though, retroactive findings indicate that just one [107] to two [108] mutations in the HA gene suffice to abolish—hence to establish as well, inversely—viral transmissibility (meaning pandemicity, in that case). Those mutations could have taken place within a pig or a man. Yet, it is not clear what earlier evolutionary path took place and eventually endowed the 1918 pandemic strain with human infectivity [109], which is a prerequisite for transmissibility. At any rate, it has been proposed that the H1N1/1918 pandemic strain was a reassortant carrying some genes from the preceding pandemic strain H2N8/1900 [89], which is presently an unrecoverable pandemic strain, and will apparently remain as such.

### 6.2. 2009 Swine H1N1 Pandemic Strain

Ninety one years after the emergence of the 1918 swine flu pandemic strain, its phylogenetic course was followed, basically, by another porcine triple reassortant pandemic virus. The latter pandemic strain represents, putatively, the second case in which the HA subtype was not substituted for by a new pandemic strain bearing a different HA subtype. The first one (Table 3) occurred in 1857, and—coincidentally or not—a similar pattern repeated in 2009, whereby a preceding Asiatic H1N1 virus (initially A/USSR/90/1977) was followed by an H1N1 virus that appeared in America. In 2009, the source was porcine; naturally, the source of the 1857 strain is unknown. Having the image of “swine flu,” though, the 2009 strain caused exaggerated fears in terms of its potential virulence. But it turned to be, thus far, a mild strain, not at all resembling the 1918 swine influenza pandemic, in that sense. Debatably, in terms of metric “Years of Life Lost” (rather than numbers of deaths), its severity was estimated to be appreciable, taking into account the particularly young age distribution of deaths and using a methodology similar to that used to generate excess mortality burden for interpandemic influenza seasons [110]. At any rate, it is a pandemic strain much less virulent than the 1918 swine pandemic strain.

Still, in similarity with the 1918 strain, here as well, the 2009 strain aroused a controversy, having an ostensibly exceptional genomic composition [111]:

- swine (North America): HA, NP, and NS;
- swine (Europe): NA;
- swine (Eurasia): M;
- avian (North America): PA and PB2;
- human (1993 H3N2 strain): PB1.

This complex intercontinental genomic composition caused Gibbs et al. [112] to suggest that “the possibility that laboratory errors involving the sharing of virus isolates and cultured cells, or perhaps vaccine production, may have been involved… It is important that the source of the new virus be found, if we wish to avoid future pandemics rather than just trying to minimize the consequences after they have emerged.” 

Yet, this pandemic strain initially formed, as a matter of fact, in pigs, in America, involving, plausibly, the above-mentioned, prime conjunction of co-farming of pigs and turkeys in Mexico, where it first emerged. It so happened that, in the USA, H1N1 subtype exclusively was prevalent among swine populations before 1998, whereas since 1998, H3N2 strains have as well been isolated from pigs (plus turkeys, and later on H1N2 strains, too). As of 2004, H3N2 virus isolates in the USA swine and turkey stocks were triple reassortants, containing genes from human (HA, NA, and PB1), swine (NS, NP, and M), and avian (PB2 and PA) lineages [80]. A deduction can readily be made regarding Mexico, due to the geographic proximity and similar pig-turkey co-farming. In that case, the H1N1 2009 pandemic swine flu virus—having an analogous genomic composition, within which porcine HA and NA (H1N1) replaced the human HA and NA (H3N2)—could have likely been an outcome of that evolving triple-interface (human-porcine-avian) genomic apparatus. Conceivably, though, such apparatus, which relies on a human-sustained swine-turkey interface—and is at times complemented by intercontinental viral gene conveyance through migratory birds and human travelers—has gradually been forming in America since the 16th century (when domestic pigs were first introduced into America), and could have given rise to both the H1N1 1857 pandemic strain and the H1N1 1918 swine flu pandemic strain, too. 

### 6.3. The Abortive 1976 H1N1 “Prepandemic” Strain

In 1976—namely, 19 years after the subtype H1N1 disappeared from human populations worldwide—another H1N1 subtype virus—A/New Jersey/11/76—reappeared within humans in the USA. The virus locally infected some 230 soldiers in a recruit camp throughout 3 weeks and then just vanished. It was characterized as a porcine strain, antigenically [113]. Further, its HA [114] and NA [115] genes were sequenced, but their concrete origins were not traced. The NP genes of two A/New Jersey/76 isolates were analyzed; one clustered with the then recent H1N1 swine viruses of the USA, and the other one with contemporary human strains [116]. The provenance of the other genes of A/New Jersey/76 is not known. Full genomic comparison of the epidemic 1976 New Jersey strain to the 1918 and 2009 pandemic swine influenza strains, as well as to sporadically human-infecting swine strains, could probably facilitate understanding of what enabled its transmissibility—as compared to recurrent nontransmissible, sporadic human-infecting porcine strains—on the one hand, and its incapability to scale up from epidemic into a pandemic virus—as compared to the 1918 and 2009 pandemic swine strains—on the other hand.

From an epidemiological viewpoint, though, a comprehensive elucidation has been presented [117]. It was thereby suggested that the epidemic virus A/New Jersey/76 ran its natural course, reaching extinction due to the depletion of susceptibles in the infected military platoons and an inability to maintain transmission outside of this favorable environment, and that there is no reason to invoke viral competition or any other extrinsic factor to explain its subsequent disappearance. Further, the virus had yet another challenge for spread outside of the platoons: the threshold for herd immunity to a virus with an R_0_ of 1.2 is 17% of the population being immune (9% for an R_0_ of 1.1). Military vaccines administered before 1969 contained swine HA antigen, and H1N1 circulated until 1957, so older personnel may have been protected against the A/New Jersey/76 strain, and immunity in even a small fraction of the population would have been adequate to halt the spread of the virus. That a virus with the odds heavily stacked against it could undergo six or more serial passages through humans is the cause for concern. That this virus did not mutate to be more transmissible in humans or reassort with the ordinarily cocirculating H3N2 A/Victoria strain may be indicative of its lack of fitness, or it may be luck. The greater is the number of serial transmissions in humans, the greater is the probability of reassortment and adaptation to humans. 

The 1976 H1N1 swine epidemic strain did not shape into a pandemic one, thereby illustrating the essentiality of a missing critical mass. It infected 230 personnel in a military base due to repeated human-to-human transmissions, over a period of three weeks. This virus was then characterized as “a strain that appears to have developed the capacity of modestly successful person-to-person transmission in the surroundings of a military recruit center but was unable to propagate itself successfully in a normal civilian population” [118], although the initial causative virus originated in a farm, outside the concerned military base. Lately, this outbreak was regarded to be “a zoonotic anomaly caused by introduction of an animal virus into a stressed population in close contact in crowded facilities during a cold winter” [119]. Under those circumstances, apparently, the above-mentioned “modestly successful person-to-person transmission” was contact transmission, rather than airborne transmission, fairly equivalent, in principle, to current familial clusters of H5N1 human cases in Asia, and to the past H7N7 epidemic in The Netherlands, as further elaborated on below.

All in all, this porcine-derived virus acquired infectivity towards but partial transmissibility within humans, hence did not make the transition from epidemicity to pandemicity. Substantial gaps remain regarding its origin, genomic profile, and inherent potency, irrespective of herd immunity. A major issue is whether the virus spread across the infected dense recruit population by merely contact transmission, or—much less likely—by airborne transmission. The latter mode of transmission is probably vital for attaining pandemic capacity [51]. Notably, the exceptional appearance of the H1N1 New Jersey strain in 1976 in America slightly preceded a series of further enigmatic H1N1 strains that emerged from 1977 to 1991 in Asia, as follows.

## 7. Further Enigmatic H1N1 Strains

### 7.1. 1977 H1N1 Pandemic

The origination of the Asiatic 1977 H1N1 pandemic strain, albeit regarded to be a direct derivative of the seasonal epidemic strain A/Fort Warren/1950, is not that plain, and is in fact unknown, in terms of the way it reappeared after 27 years (and 20 years after the H1N1 subtype was globally replaced by H2N2, in 1957). It was designated A/USSR/90/77, although it surfaced in Anshan, China. The event was a remarkable mystery. It has been suggested that this virus was kept in some lab, from which it somehow got free [120]. This is a plausible and trivial possibility, but it would be reasonable to assume that, during the 34 years that passed since 1977, the concerned lab or institution would have been aware of such a meaningful biological anomaly and openly disclose this affair. Anyway, virus leakage is of course a possible explanation, yet certainly not the sole one, particularly that the strain A/USSR/90/77 was found to actually be a reassortant virus, of which the gene segment coding for the M protein displays rather considerable homology to the corresponding gene of an earlier human epidemic strain—A/FM/1/1947 [121]. Besides, specifically China, once again—reckoned as a prime natural provenance of pandemic strains at large—is where the 1977 H1N1 virus surfaced. The mentioned reassortant virus could have been formed, basically, in various fashions, both artificial and natural. One mechanism that could be involved in its perennial gene conservation is preservation in environmental ice of arctic lakes visited by migratory waterfowl [122]. Referring to at least certain gene segments that are shared by both avian and humane strains [123]—as well as to any gene segment or entire genome included in the enormous pool of AIVs, overall—this appears to be a feasible mechanism. 

Geographically, the 1977 dually characterized Russian-Chinese H1N1 pandemic strain likely originated in Mongolia. Bridging between China and Siberia—and thus forming, altogether, the cardinal Chinese-Mongolian-Siberian pathway of migratory waterfowl—the land of Mongolia may be regarded as a prime ecogenetic melting pot of influenza A viruses. In the northwest of Mongolia, tucked between the Altai, the Hangayn, and the mountains of the frontier with Russian Siberia, lies a scenic basin complex known as the Great Lakes region, in which are strewn more than 300 lakes [124]. Those lakes, as well as other lakes in the mountainous areas of Northern Mongolia, are abundantly visited by waterfowl during summer [125]. This region appears to constitute a paramount influenza A virus nidus. Uniquely to Northern Mongolia, and not casually, so it seems, this Lake Baikal-bordering region is populated by two outstanding wild mammals, as well: the Mongolian wild horse (*Equus ferus*—same species as the domestic horse), which is presently the only true wild horse worldwide, and the Baikal seal (*Pusa sibirica*)—the only true freshwater seal in the world. Notably, antibodies to H3 HAs of the human H3N2 strains A/Aichi/2/1968 and A/Bangkok/1/1979 were detected in Baikal seals [126]. Horses and seals are among the numbered mammals that are influenza A virus hosts. Therefore, avian-mammalian interfaces involving influenza viruses (and presumably amplified by colocated wild boars) most probably take place thereupon. The mentioned Mongolian lakes are frozen during wintertime, and the higher their location is, the longer is the longevity of ice, at times perennial. Perennially frozen lakes are found, as well, in nonmountainous islands located in the Arctic Ocean, extending, thus, the range of the waterfowl northerly migration axes. Whenever getting thawed and until refreezing occurs, the lakes are immensely occupied by breeding waterfowl, which are prone to contract viable influenza viruses released by melting ice. Those viruses might contain genes of past pandemic and seasonal strains, and are predisposed to be recirculated.

### 7.2. Subsequent Obscure H1N1 Strains in Mongolia

Peculiarly, the land of Mongolia gave rise to further enigmatic H1N1 strains, from 1979 to 1991. In the autumn of 1979, a severe influenza epizootic started, quite exceptionally, among camels in Mongolia [127]. Between 1980 and 1983, 13 independent isolates of H1N1 viruses were obtained from diseased camels and were virtually indistinguishable from the pandemic prototype A/USSR/90/77 strain, serologically. Two hundred and seventy-one samples of camel sera collected between 1979 and 1983 contained antibodies against the A/USSR/90/77 isolate. After experimental infection of camels with some of these isolates, the animals developed similar symptoms as those found during natural infection: coughing, bronchitis, fever, and discharge from nose and eyes. A genetic sequence analysis revealed that among the eight gene segments the PB1, HA, and NA genes of the camel isolates were almost identical with allelic genes of A/USSR/90/77, and the PB2, PA, NP, M, and NS genes were almost identical with those of the human A/PR/8/34 strain [128].

Following the morbidity within camels, lingering human morbidity occurred. Four epidemic influenza A viruses of the subtype H1N1, isolated from Mongolian patients between 1985 and 1991, were analyzed by sequencing of various RNA segments. An isolate from 1985 was found to be highly related in all genes sequenced to strains isolated from camels in the same region and at about the same time [129]. Interestingly, it has been suggested that these camel isolates were presumably derived from a human UV-light inactivated reassortant vaccine (A/PR8/1934xA/USSR/1977) prepared in Leningrad in 1978 and used in the Mongolian population at that time. Yet, a human isolate from 1988 was also found to be a derivative of a reassortant between PR8/1934 and USSR/1977, but in contrast to the 1985 isolate it contained an HA closely related to A/PR8/1934. Further, one of the isolates from 1991 was in all genes sequenced and found closely related to A/PR8/1934, while another isolate from 1991 was closely related to H1N1 strains isolated around 1986 in other parts of the world. It was observed that the mutational and evolutionary rates of the Mongolian strains seem to be significantly lower when compared to the rates of human influenza A strains isolated in other parts of the world, and that viruses might thereby keep the potential to reappear in the human population after years, bringing about a pandemic, as was the case in 1977. 

Nevertheless, it follows that various Mongolian mammalian isolates or part of their genes have been somehow conserved for decades and then resurfaced. This may happen due to some human intervention, or preservation in environmental ice, from which released viruses—upon its melting—can be contracted and consequently disseminated by waterfowl. Regardless of the enigmatic 1977 pandemic strain, all the known other genomes of pandemic strains consistently include genes of waterfowl influenza viruses. In case natural abiotic preservation of influenza A genes—mammalian and/or avian—does take place in ice, it might contribute genes of past pandemic and seasonal strains to emerging ones. If such, or closely related, past genes—particularly encoding for the HA or NA antigens, or for certain internal proteins (as elaborated on below)—are involved, they can give rise to potentially prepandemic strains, subsequent to being contracted, reassorted, and conveyed by migratory waterfowl.

## 8. The Paramountcy of Avian Influenza Strains

Fundamentally, influenza type A viruses are lastingly entrenched among waterfowl. In terms of virus ecogenetics, the primary animal host populations constituting the melting pot predisposed to create critical mass for resultant formation of a pandemic strain include wild waterfowl (ducks, geese, and swans), domestic waterfowl, pigs, chickens, turkeys, and horses. The order they are presented reflects the level of overall viral genetic dynamics marking them, hence the likelihood of each serving as a productive genetic melting pot, in descending importance. Also, the intensity of direct interface between those animal populations and man poses an additional factor—not following the same order—that is appreciably influential as both a vectorial (allowing avian/mammalian-to-human virus transmission) and a productive (allowing formation of new viral genotypes) critical mass. Further host species—such as quails, gulls, seals, dogs, and cats—are as well involved in similar manners, though apparently but collaterally. Duck populations, both wild and domestic, are of paramount importance, because they constitute the most permissive host, hence minimally affected by potent—exclusively avian, as well as premammalian and, occasionally, mammalian—virus strains, which they circulate and proliferate, usually asymptomatically.

### 8.1. The Avian-Mammalian Interface

Significantly, while transmission of influenza A viruses from avian hosts to mammals often takes place, as discussed above, the converse course from mammals to birds is much less observed. Moreover, the latter has been found to occur in America only, involving virus transmissions from pig-to-turkey (regularly), human-to-turkey (rarely), pig-to-quail (exceptionally, as far as presently known), and pig-to-wild duck (exceptionally, as far as known) [130].

Natural transfections from mammals to birds have commonly been evidenced from pigs to turkeys, including the subtypes H1N1 [131], H1N2, and H3N2 [132]. But at least in two episodes, rather more meaningful, virus transfections from pigs to wild ducks took place, as well. In the first episode [133], a porcine strain of the H1N2 subtype subclinically infecting a wild duck was found to be a triple reassortant, bearing avian PA and PB2 genes (like the recent pandemic H1N1 strain), plus human NA and PB1 genes, while all the rest of the genes are porcine originated. This genomic composition is basically similar to H1N2 swine flu viruses sporadically infecting humans during recent years. In a second episode [134], again, a porcine triple reassortant, yet of another, prevalent human subtype—H3N2—was obtained from healthy mallard and pintail ducks. Remarkably, this virus had genes from humans—HA, NA, and PB1; swine—M, NS, and NP; birds—PA and PB2. Rather similarly patterned, the latter reassortant virus—H3N2—differs from the former one only in that its HA gene is human derived, while the former H1N2 reassortant virus bears a swine-derived HA gene. It is of note that the same genomic patterns apply for the porcine H3N2 and H1N2 (plus H1N1) strains that are known to transmit from pigs to turkeys.

Nonetheless, those sole findings concerning intact porcine strains harbored by wild ducks are marked, in that they possibly reflect an as-yet inadequately noticed mode of transmission in nature, which perhaps follows an ultimate avian-porcine-human genomic pattern and thus substantially contributes to the evolutionary dynamics of poultry, porcine, and human—including pandemic—viral strains, altogether. Irrespectively, though, it was a sheer avian-human genomic interface, which gave rise to the H2N2 (in 1957) and H3N2 (in 1968) pandemic strains, following, still, the mentioned patterns, in that the H2N2 strain had avian PB1, HA, NA genes, and the H3N2 strain had avian PB1 and HA genes (while the rest of the genes were all human, in those two cases).

Actually, each of the genomically identified pandemic strains (of 1918, 1957, 1968, and 2009) has some avian genes. This fact chiefly represents the avian-human genetic interface of influenza type A viruses in general, and within the context of emerging pandemic strains in particular. It has been evidenced that this interface is of paramount importance [44]. Multiple episodes have been recorded, whereby fully genuine AIVs directly transmitted from birds to humans, thus demonstrating infectivity towards man, but none of those viruses were or became (through mutations) aerogenically transmissible among humans. Therefore, it seems, so far, as if an entirely innate genome of any avian influenza strain—even if infective to man—is inherently deprived of pandemic capacity.

Far and away, the avian-mammalian influenza interface is consequentially pronounced in the marine arena, too. The subtypes H13N2 and H13N9, isolated from a pilot whale (*Globicephala melaena*), exhibited close relatedness to H13 influenza viruses from gulls, which probably originated the whale isolates [135]. Another influenza virus isolated from a whale—H1N3—was found to be closely related to an isolate obtained from a human being, albeit on a singular occasion [97]. Similarly, however, in several cases, sporadic human infections were shown to be accounted for by H7N7 viruses contracted from seals [136], which likely had derived as well from gulls or other sea birds. Avian-originated H3N3, H4N5, and H4N6 viruses were also isolated from seals. Although uncertainly contributive to the genesis of pandemic strains, this avian-mammalian marine interface is notable.

### 8.2. Highly Pathogenic Avian Influenza Viruses

Highly pathogenic avian influenza (HPAI), or, as it was termed originally, “fowl plague,” was initially recognized as an infectious disease of birds within chickens in Italy, in 1878. Although Centanni and Savonuzzi, in 1902, identified a filterable agent responsible for causing the disease [137]—actually the first influenza virus ever isolated, just several years after the very first animal virus, foot and mouth disease, was isolated—it was not before 1955 that Schäfer characterized this agent as influenza A virus [138].

Among the 16 HA subtypes of AIVs, H5 and H7 have molecular traits that at times afford them with remarkable virulence towards birds—particularly poultry—and occasionally with infectivity towards humans. Thus, HPAI viruses are characterized as such owing to their ability to cause systemic, usually fatal, and markedly contagious infection in chickens. Frequently, they have the same effect within turkeys, but their virulence considerably varies within ducks and geese, both domestic and wild. By definition, HPAI viruses are solely affiliated with the subtypes H5 and H7, although sometimes they exhibit less virulence than different antigenic subtypes, even within chickens, due to the complexity of viral pathogenicity. Also, many variants of H5 and H7 viruses defined as LPAI are very common within wild waterfowl populations. Up to now, H5 and H7 subtypes were not involved in the formation of the antigenically identified pandemic strains, although avian H5- and H7-bearing viruses could have been, in principle, the donators of polymerase genes to the formations of the 1918 and 2009 H1N1 pandemic strains (Table 3). On many occasions, H5 and H7 viruses did infect man and other mammals naturally, but only within horses have H7 viruses been evidenced to regularly spread in an epidemic manner.

#### 8.2.1. H5N1—A Persistent Avian Test Case Virus of Pandemic Potential

The semianthropophilic avian highly pathogenic H5N1 virus (as well as few other viruses, such as the LPAI H9N2 or the porcine H2N3 viruses) can serve as an appropriate model case to explore the factual unrealization—thus far—of the tentatively above observed patterns of emergence of those pandemic strains that did arise in actuality and thereby point at critical masses which would have shaped the H5N1 virus into a pandemic strain, but have not formed, for the time being [51]. Typically an avian virus, perhaps only 2 amino acid changes in its receptor binding site were needed, yet, so as to change the tropism of the H5N1 HA from avian- to human-type receptors, and thereby endowed it with infectivity towards man [139]. Unprecedentedly (as far as known), the H5N1 virus is sporadically but continuously infecting humans, with remarkable fatality rate—62%—the highest recorded for human influenza viruses, and an extreme one for pathogens at large. Interestingly, and apart from Asia, where it reached the highest morbidity and mortality rates worldwide in Indonesia—with 83% fatality rate—the second country most afflicted by this virus is, outstandingly, an African one—Egypt—yet with low fatality of 48%, for unclear reasons. The H5N1 virus is exceptionally invasive in humans (and poultry), infects the brain in addition to other various organs, and might thus possibly even bring about Alzheimer's and Parkinson's disease, in the long run [140]. At the same time, it is notable that an appreciable part of humans that contracted the HPAI H5N1 virus were infected subclinically.

The virus first appeared in 1997 in Hong Kong and is presently found across the Eastern Hemisphere, threatening to invade the Western Hemisphere, as well as to transit into a pandemic strain. It became endemic in Southeast Asian countries, plus—significantly, rather than incidentally—merely Egypt, worldwide, for now. Actually, since its initial emergence, in 1997, great concern aroused that it might turn into a pandemic strain. It has repeatedly been observed that such a transition is a matter of time. Concurrently, the very fact that during the last 14 years—seemingly a considerable amount of time—such transition did not occur gave rise to an opposite approach, reckoning the H5N1 virus as being incapable of thus transforming. Each of those two contrasting schools appears to rely on a distinct rationale, yet this intriguing dichotomy is rather theoretical, because it is practically unknown what the duration of the evolutionary phase that precedes the surfacing of a new pandemic strain is. During those 14 years, in spite of current influx of multiple genetic variants furnished by the avian genomic pool of H5N1 viruses and albeit current interface that takes place between a portion of those variants and the human H3N2 plus H1N1 diversified genomic pool within humans infected by H5N1 strains, the latter did not acquire airborne transmissibility among humans and therefore did not become pandemic. 

At least on several occasions, the H5N1 virus has conclusively been observed—in spite of its marked virulence—to be disseminated in nature by subclinically infected migratory waterfowl [141]. Connectedly, the essentiality of the Siberian-Chinese biogeographic axis—an axis superfluously occupied by migratory waterfowl—for the generation of human-adapted and potentially pandemic strains is one main element in the present analysis. It is further accentuated by the fact that the geographic origins of most of the traceable pandemic strains were either Russia or China. The likely provenance of the strain A/teal/Hong-Kong/W312/97, which is the chief progenitor of the human prototype HPAI H5N1 A/Hong-Kong/97, has been observed to be Siberian lakes [142]. Southerly migrating teals, mallards, and many other ducks maintain close interface with free-grazing domestic ducks in rice paddies, altogether continuously perpetuating the HPAI H5N1 virus [143]. All in all, the prominent contribution of domestic ducks to the environmental endurance of the HPAI H5N1 virus, primarily in Southeast Asia, has been pointed at [144, 145]. Colocated migratory ducks and domestic pigs fuel and amplify, allover, the genetic dynamic of the H5N1 virus, in conjunction with other influenza A viruses. 

Vast outbreaks in poultry, mainly in chickens, caused by HPAI H5N1 strains in Republic of Korea and Japan during 2007 suggested that migratory waterfowl could indeed be a strong mediator for the spread of the HPAI H5N1 virus in Southeast Asia. [146]. In addition, the role of wild ducks, geese, and swans as spreaders of HPAI H5N1 viruses from Asia onto Europe has been evidenced [147]. The road to America, although seemingly less probable for now, may take shape via the Bering Sea, across the Atlantic Ocean [148], and through the regular, close interface taking place each summer between European and American waterfowl that occupy the same lakes in Greenland, not too far from Canada [149]. Relatedly, the importance of Canadian ducks as critical mass shapers is potentially equivalent to that of Asian ones, in principle. They are heavily infected by multiple LPAI strains [150], and their particular role in the extensive formation of genetic reassortant influenza viruses has been pointed out. Also, H5N1 outbreaks in downwind areas of Asian dust storms suggest that viruses might as well be transported by dust storms, which are becoming across Asia longer and more frequent as a result of desertification in China. Viruses attached to particles of dust stirred up by these storms can potentially travel long distances and occasionally reach as far as Europe or the USA [151].

Thorough findings regarding the evolution of H5N1 influenza viruses in domestic ducks in Southern China imply that the human H5N1 A/Hong Kong/97 prototype evolved, initially, in ducks and that some of its genes reemerged or persisted almost unchanged in ducks, until at least the year 2000; moreover, phylogenic homogeneity of the HA genes of multiple duck-originated isolates has thereby been observed from 1997 until at least 2002 [152]. Even far beyond, the entire genomes of viruses isolated in 2005 from shell washes of duck and goose eggs from Vietnam were found to be practically identical to the human prototype A/Hong Kong/97, whereas no explanation could be offered with respect to their virtual unchangeability from 1997 until 2005 [153]. This unchangeability is surprising, ostensibly, considering the remarkably rapid evolutionary dynamics of various influenza A subtypes among wild and domestic aquatic avian host species at large (>10^−3^ substitutions per site, per year) [26]. But this unchangeability is highly consistent with the postulation that abiotic preservation of influenza A viruses can readily take place in Siberian (and Mongolian) lake ice [122, 154]. At any rate, the exclusive, conservative endurance of the H5N1 A/Hong Kong/97 prototype lineage may be regarded, foremost, as a potential critical mass for a tentative genesis mechanism of a pandemic strain. Conceivably, then, such a conservative, yet ultimately constructive viral endurance is shaped by wild waterfowls through their interface with lake meltwater during summer (in Siberia and Mongolia) and through their interface with domestic waterfowl during winter (in China and Southeast Asia). The periodicity fashioned by this apparatus could be annual, as well as perennial, depending on ice longevity. Migratory waterfowl, the primary hosts of influenza A viruses, would anyway reach, every summer, the most northerly line of meltwater and occupy it.

Although on many occasions HPAI H5N1 avian-to-human transmission—either respiratory or nonrespiratory—has indeed been conclusively reported [155, 156], human-to-human transmission occurring thereafter [47] does not reflect prepandemic critical mass, as long as not airborne. Therefore, H5N1 human-to-human transmission is practically meaningless, unless taking place aerogenically. Simply stated, “low-level human-to-human transmission is not necessarily indicative of an emerging pandemic” [156], whereas the converse formulation expressing an airborne transmissibility-dependent critical mass, as posed by the WHO, refers to “sustained or community human-to-human transmission.” Also, cohort studies found that human-to-human transmission might have occurred through close physical contact, but not through social contact [157]. This characteristic reflects, indeed, the distinction between direct and airborne transmission, respectively. For now, the latter has not taken shape, with regard to the HPAI H5N1 virus. 

This cruciality was demonstrated in Indonesia in a remarkable manner. A cluster of eight H5N1 flu cases within an extended family was thoroughly analyzed and found to include seven person-to-person transmissions, due to “sustaining close contact with other ill family members prior to getting sick.” Nevertheless, the in effect mode of those transmissions has not been inquired into [158]. Indonesia's Health Minister commented, though, that the research findings had “misled the public. If there had been human-to-human transmission, it would have already swept the country and killed thousands. Our scientists have already determined that the 2006 outbreak in North-Sumatra was not a case of human-to-human transmission” [159]. By all means, no airborne human-to-human transmissions took place during that event, but most probably sheer human-to-human contact transmission. Clusters representing the same pattern, basically, occurred in China, Egypt, Azerbaijan, Iraq, Turkey, and Pakistan. Thus, instantly, one family cluster in China has been characterized as “substantial unprotected close exposure” that yielded “probable limited person-to-person transmission” [160], namely, sheer contact transmission. Has an aerogenically transmitted H5N1 strain caused those clusters, it would have generated, in all likelihood, a critical mass triggering a pandemic.

Eventually, the H5N1 virus brought about a conceptual shift with respect to the genetic dynamics of influenza A viruses. Different from the widely accepted approach that avian viral strains have the capacity to infect man only after undergoing genetic reassortment within pigs, it is now contended that direct transfection of man by intact avian-harbored viral genotypes is an actual, recurrent move. This cardinal shift ostensibly reflected a genuine, unprecedented path within the evolutionary paradigm of Influenza A virus. It has been suggested, though, that direct avian-human genetic interface is a pristine fundamental within the natural history of influenza A viruses, and that human infectivity of the H5N1 virus (and alike) is a readily detectable and traceable phenomenon, presently, rather than representation of a novel development [44]. The direct avian-human genetic interface could certainly account for the creation of pandemic strains, like H2N2 in 1957 and H3N2 in 1968. 

#### 8.2.2. An Avian H7N7 Virus Generating a Threshold Epidemic in Humans

Rather symbolically, in a sense, the first AIV ever isolated from a human being (in the USA, in 1959), resembled, antigenically, the first influenza virus ever isolated at large (in Italy, in 1902), which was called “Fowl Plague” virus—a pathogen that virulently proliferated in chickens [161]. Both viruses were HPAI of the antigenic subtype H7N7. Also, different from the ongoing lingering, endemic, yet mainly sporadic course marking the HPAI H5N1 virus occurrences within humans in Asia and Egypt, only in one additional case an avian HPAI virus—on that occasion H7N7—attracted extraordinary attention (in 2003, in The Netherlands), singularly causing a short-term, though fairly extensive outbreak among humans. Still, even in that case, too, the virus was not aerogenically transmissible among humans, apparently, not having, therefore, pandemic capacity, and just vanished, consequently [162].

Lasting—in terms of documented clinical cases—for several weeks, this epidemic event involved 89 human cases who handled avian influenza-affected chickens and three of their family members, with one fatal case. During that outbreak, the causative agent—an innately duck-originated influenza strain of the subtype H7N7—exhibited notable infectivity while passing from chickens to humans. Moreover, serologically, it was estimated that virus infection occurred in 1000 to 2000 people and that person to person transmission may have taken place on a large scale. Nevertheless, the genetic alterations needed to form the critical mass yielding effectual viral airborne communicability did not occur. Representing a crucial threshold condition, this outstandingly significant outbreak arrived quite soon, resultantly, at a dead end, reminding, thus, of the 1976 New Jersey swine abortive epidemic. They both differ, hence, from the still ongoing, sporadic, yet lingering human morbidity caused by the present HPAI H5N1 virus, but they all similarly illustrate the lack of a transition to a fully, aerogenically communicable pathogen. The H7N7 and H5N1 scenarios clearly demonstrate, however, the potential of LPAI viruses to cross an interavian species barrier, mutate into HPAI, and then cross an additional, rather crucial species barrier, which exists between avian and mammalian—including human—host species. Conceivably, the greater the phylogenetic distance among the involved host species is, the more significant are the crossed species barriers.

Remarkably, the H7N7 subtype (aside H5N1) is the most prevalent antigenic subtype among the HPAI viruses isolated throughout the past 109 years from poultry worldwide [163], implying the potency of that specific antigenic combination. High genomic identity of the HPAI H7N7 virus that caused the epidemic in 2003 (subsequent to outbreaks in chickens) to low pathogenic—probably precursor—viruses isolated from wild ducks has been evidenced [162]. On the whole, like the current HPAI H5N1 virus, the HPAI H7N7 under discussion illustratively reflects the shift from a benign virus circulating in wild waterfowl—such of which are numerously found in nature—into a virulent pathogen that severely inflicted poultry, and thereafter passed on to man, threatening to propel a pandemic. This short-term, but plainly evolving chain of infections may significantly well resemble an ancient, equivalent yet long-term, gradually gathering course, throughout which took place the primal evolution and emergence of prepandemic strains from ordinary, wild duck-harbored LPAI viruses, thus creating the crucial platform for the next, ultimate step—the genesis of a primordial pandemic strain. Porcine involvement in that ultimate step could have occurred, but was not necessary.

### 8.3. Infections of Humans by Avian Strains of Low Pathogenicity

Some benign avian strains can as well infect man, directly. Another episode involving a natural human infection with an avian strain took place in England in 1995, once again due to H7N7—yet this time a LPAI variant, and a much more concrete one, in terms of the source of infection. It appears that the latter episode properly reflects a prime mode of virus conveyance, in that the individual human affected by the virus contracted it from pet ducks sharing a lake with migratory birds [164]. Yet the clinical manifestations of both the described HPAI and LPAI H7N7 strains were relatively mild in humans—conjunctivitis merely (in 1995—LPAI strain), associated with transient respiratory illness (in 2003—HPAI strain). Additional 6 episodes of natural human infections involving the H7 HA were recorded worldwide—distinctively in the UK, USA, and Canada—all due to low pathogenic strains bearing N2 or N3 NA, which caused conjunctivitis and transient respiratory illness. Overall, the significance of the H7 subtype as a potential source for pandemic strains has been accentuated [136].

Other AIVs that happened to naturally infect man belong merely to the LPAI subtypes H9N2 and H10N7. While the H9N2 virus became endemic in poultry in Asia and accounted for several episodes of sporadic human infections, the H10N7 has been isolated but once from a human being, in that case, yet, in Egypt. Also, antibodies to AIVs have been reported in humans in Southern China; these antibodies were detected by single radial hemolysis, an assay that is insensitive to inhibitors. Interestingly, the highest seropositive reactions were for the innately avian H11, H6, and H4 subtypes, with 15%, 12%, and 11%, respectively [165]. Such seemingly random occurrences of AIVs within humans have been inquired into by experimental infections of volunteers. While the direct transmission of influenza viruses between human and swine is frequent and fully evident, parallel interface involving bird-to-human transfections is not as plain, though potentially of noticeable importance. Therefore, human experimental infections—albeit fairly rare, in general—were conducted, so as to comprehend this interface. Due to the singularity of those human experimental infections, a summary of the results is herewith presented. 

Eight influenza subtypes isolated from ducks, mostly, and turkeys, were used, representing the then known HA subtypes, except for the potentially highly pathogenic H5 and H7, the pandemic-resembling H2 subtype, and the benign avian subtype H8. The NA subtypes used represented all those already known to infect humans, namely, the pandemic N1, N2, and N8 subtypes and the sporadic N7 one. Marked infectivity rates were obtained—17–50%, with a single exception of H6N2—though it should be noted that high-dose challenges were applied, thus increasing the prospects for productive infection. Notably, H4 and one of the two H6 viruses tested—all representing subtypes which have never been observed to be infective to man—did propel infection and, moreover, generated clinical symptoms (alongside with H9N2) [48]. The main findings are herewith presented, due to their singularity (Table 4).

Those findings, together with the mentioned findings of human seropositivity for LPAI viruses, are indicative of appreciable human infectivity of those viruses, further to the mentioned natural infections of man by HPAI, and additional LPAI viruses. Both LPAI and HPAI viruses are capable, so, of donating genes to emerging pandemic strains, even if the latter form within humans only. Connectedly, it has been suggested that if there is epidemiologically significant variation among avian influenza virus genotypes, then evolving of a pandemic strain is more likely to take place due to avian virus outbreaks stemming from repeated cross-species transmission events than those caused by low-level transmission between humans [166].

## 9. Observations Pertaining to Antigens, Genes, and Genomes

### 9.1. Antigenically Based Observations

Since 1847, 10 pandemics have occurred, reputedly involving 5 different antigenic subtypes (Table 3). Eight additional antigenic subtypes, mostly avian, were isolated sporadically or singularly from humans since 1959, with the exception of one, short-term epidemic episode generated by an avian H7N7 virus (Table 5). Further avian antigenic subtypes exhibited infectivity towards man indirectly (detected serologically, merely), as well as by means of experimental infections.

While all H1, H2, and H3 pandemic strains (since 1918, excluding the exceptional 1977 H1N1 strain) contain genes from both mammalian and avian origin, the nonpandemic, H5, H7, H9, and H10 strains are fully avian derived. Also, a prominent feature of the identified pandemic strains (since 1847) is that they include only 3 HA subtypes out of 16 HA subtypes, and only 3 out of 9 NA subtypes presently known to generally exist. Moreover, each of the pandemic HAs and NAs recycled, one way or another (Table 3). These reoccurrences are most probably not casual, then, reflecting innate affinity of those 6 viral surface proteins towards the human host, in terms of both infectivity and airborne transmissibility. Further, the 10 emergences of the antigenically identified pandemic strains represent 5 combinations of the concerned HAs and NAs, out of 9 possible combinations. All the other 4 antigenic combinations most probably formed within human populations, but did not persist due to inadequate survival value. A considerable degree of compatibility between the HA and NA molecules has to prevail within a given virus [123], so as to allow for their synergistic functioning in a given host species. Such compatibility (in conjunction with certain additional viral proteins) may well enable infectivity, but not necessarily transmissibility (which is vital for pandemicity); therefore, it may be observed that the additional 4 antigenic combinations, though in all likelihood shaped over time, were apparently unproductive, in that sense. Deductively, antigenic combinations other than the 5 pandemic-representing ones, particularly including HA or NA subtypes different from the pandemic three HAs and three NAs mentioned—albeit not excludable—are inherently appreciably less capable, if not completely incompetent, of giving rise to pandemic strains.

Among the nonpandemic antigenic subtypes, H7 is clearly the most common one, and although potentially a highly pathogenic subtype, it but mildly manifested itself clinically upon contraction by humans and did not persist for long. By contrast, the other potentially highly pathogenic subtype, H5, emerged only once as a human pathogen (H5N1), but does manifest itself as an extremely virulent virus, and persists for about a decade in Southeast Asia and Egypt. At the same time, H7 (in the form of H7N7 equine influenza, particularly) already proved full adaptability, including complete transmissibility, in a mammalian host. It would be too speculative, though, to evaluate whether an H5 or an H7 virus is more—if at all—liable to emerge as a pandemic strain. All in all, comprising, potentially, the two mere highly pathogenic subtypes (as defined for chickens, originally), H5 and H7 consist half of the 4 non-pandemic subtypes isolated from naturally infected humans, thus far, alongside 3 pandemic subtypes (H1, H2, H3), and 9 HA subtypes that have not been isolated from humans, for now.

Also notable is the versatile potency of N2, in that it gave rise to two different pandemic subtypes—H2N2, as well as H3N2—and three different non-pandemic subtypes (Table 5); N8 gave rise to two pandemic equivalent subtypes—H2N8 and H3N8—merely; N1 and H1 combine only with one another in the form of a pandemic subtype, while H1 gave rise to two and N1 to one non-pandemic subtypes, N7 to two non-pandemic subtypes, and N3 to two nonpandemic subtypes.

### 9.2. Genetically Based Observations

Various avian genes lend considerable, if not critical, input into the formation of pandemic strains. Such involvement is allowed for by genetic reassortment or recombination processes, which occur within rather nonavian, meaning mammalian hosts, mainly pigs and humans, and enable concomitant input by contemporary porcine and human influenza viruses. At the same time, it appears as if just few mutations—though very specific and not fully recognized—might be required and suffice for certain porcine strains (harboring swine, human and avian genes) which already gained infectivity towards man, to become aerogenically transmissible among humans, hence pandemic. By contrast, innately avian strains which gained infectivity towards man are probably incapable of undergoing such purely mutational transition. 

Genomic analyses of the last four pandemic strains (irrespective of the controversial 1977 H1N1 strain, as already discussed) show that the genes contributed by avian strains, on the whole, are those that encode for all and only the polymerases and surface antigens, as follows from Table 6. Inversely, the NP, NS and M genes are persistently mammalian.

Clearly, the input of avian polymerase genes is essential, referring to those four cases. Yet, it is apparent that while both avian and swine viruses were involved in the creation of pandemic strains—in 1918 and 2009—the genes contributed in those two cases by avian strains did not include genes that encode for surface antigen(s), whereas the creations of the pandemic strains that did not involve swine viruses (in 1957 and 1968) were facilitated by contribution of both polymerase (PB1) and surface antigen avian-genes. Also, the PA plus PB2 (along with the rest of the internal genes) in 1957 and 1968 strains are persistently human. Although all those four genomically profiled examples do represent avian gene-harboring pandemic stains, they constitute but a small portion of the history of pandemic influenza at large; it would be reasonable, nevertheless, to infer that the contribution of avian genes for the emergence of pandemic strains has been vital since the very far past (Table 1). Still, a corollary open question is whether viruses containing one or more of the NP, NS, and M gene segments from an avian strain are inherently deprived of pandemic capacity. This might be the case, referring to the existing non-experimental data, while experimental infections within a wide spectrum of model animals, including ferrets and monkeys, showed considerable genotypic variability [167], which may doubtfully reflect human infectivity and transmissibility per se. 

Not less important are the contraction and circulation of genomes or genes from pandemic and seasonal human strains by avian species. Genomes or genes of such human strains, known to have been contracted by avian hosts, either naturally or experimentally, are depicted in Table 7.

Partial reciprocality between the data included in Tables 6 and 7 does exist, particularly at the level of individual genes. Naturally, no gene circulation can take place, as long as the genotypes bearing it are infective but not communicable. However, thanks to ongoing genetic reassortment events, any of the involved genes may potentially be incorporated into an emerging communicable genotype. While viral genotypic dynamics extensiveness and intensiveness are fairly evident in aquatic ecosystems, the situation in terrestrial ecosystems is largely unclear. Aside from the important role played by feral and wild boars in that concern, pikas in Asia (China), raccoons in America (USA), and possibly other mammals may be contributive as well, but the role of wild landfowls—specifically junglefowl, turkey, and quail—although affiliated with the same species of the widely infected equivalent poultry, is unknown, if any. Basically, nonetheless, any gene segment of any human strain might tentatively reach the ongoing avian, mammalian, aquatic, or terrestrial gene pools of influenza A virus and retain potentiality to later on possibly be reassorted into a newly evolving pre-pandemic strain.

Altogether, the above presented data and multiaspect observations are here regarded as such that they could underlie the apparatus responsible for the genesis of primordial and late pandemic strains, thereby facilitating the comprehension of the mechanisms prone to generate future pandemic strains. Certain regularities that have connectedly been configured appear to be met, while a tangible degree of plasticity prevails alongside. The natural history of complex interfaces involving man, wild animals, domestic animals, and feral animals has thus been broadly elucidated in the present study. Fundamentally, the domestication of certain avian and mammalian species formed, as suggested in an interdisciplinary manner, an apparatus that brought about the earliest genesis of pandemic influenza strains, is currently operating—supposedly based on the same pristine principles—and is apt to likewise give rise to further pandemic strains. Biogeographic, antigenic, and genomic patterns accounting for that apparatus are consequentially outlined and specified, as follows.

## 10. Principal Inferences

In spite of various uncertainties that are inevitably included in the present analysis, essential, if approximate, inferences can be arrived at, through which common denominators as well as variances underlying the modes of emergence of both ancient and recent pandemic influenza strains from viral reservoir in animals are pointed out. The following inferences are based, then, upon the data presented in the previous chapters of this study.

### 10.1. Principal Inferences at the Biogeographic Level

- Formation of pandemic strains in the Northern Hemisphere is much more likely than in the Southern Hemisphere, probably due to the complex, multifactorial dynamic ecology of influenza A viruses, which is largely affected by a variety of geoanthropologic and zoogeographic elements. Still, the possibility of such formation in the Southern Hemisphere is not negligible.
- Except for the purportedly single origination of the 1510 pandemic in Africa, the recognized pandemic strains formed in Asia, mostly, America (North and Central), occasionally, and perhaps Europe (Russia, altogether, including its European part). An Asian-American interface apparently preceded the emergence in America of the pandemic strains of 1761, 1857, 1918, and 2009.
- To an appreciable degree, those two principal geographic axes (Asiatic and American) overlap with prime seasonal migration pathways of waterfowl populations, portions of which are consequentially adjacent to poultry. Certain migratory aquatic birds—in addition to humans—account for the main viral interface between the two axes.
- Along the eastern axis, the prevalent combination of the concerned domestic animals includes pigs, chickens, and ducks, whereas along the western axis the analogous combination includes pigs, chickens, and turkeys.
- The latter distinction probably fuels east-west dissimilarities within the viral ecogenetic dynamics underlying the formation of pandemic strains. Those dissimilarities are amplified by different local wild avian faunas, but are partially counterbalanced by major holarctic waterfowl species, like the mallard (which in that sense compensates for the paucity of domestic ducks—same species, *Anas platyrhynchos*—in America), and by waterfowl-shaped inter-hemispheric gene conveyance (as is, e.g., the case with the two Eurasian-originated genes of the recent, primarily Mexican swine-derived pandemic strain).
- Particularly in China and Southeast Asia, viral spatio-temporal gene and genome flow within avian faunas is largely facilitated in that interspecies barriers are prevalently bypassed through the adjacency of domestic, feral, and wild waterfowl affiliated with the same species, namely, mallard, grey goose (*Anser cygnoides*), and whooper swan (*Cygnus cygnus*); significant, too, is the equivalent bypass taking place through domestic, feral, and wild pigs, worldwide. Intergenus barriers, albeit naturally greater, are yet commonly bypassed too, for instance through transfection between mallard and pintail (*Anas acuta*), or mallard and teal (*Anas cecca*).
- Chronologically, it appears that the initial domestication and ongoing farming of pigs in China could not have enabled alone the primal evolvement of pandemic strains during about 3000 years, whereas co-farming of pigs with chickens, and then, in particular, with domesticated mallards, did propel that process. Neither likewise, apparently, could the initial domestication and ongoing farming of turkeys in Mexico during at least 2500 years, until the introduction of domestic pigs from Europe to Mexico and consequent co-farming took place.
- The viral ecophylogenetic attributes of the Eastern Hemisphere, as a whole, yielded avian-originated pandemic strains (H2N2, H3N2), a self-limited avian-originated epidemic strain (H7N7, The Netherlands), and a lasting avian-originated human-infecting endemic strain (H5N1), all bearing no porcine genes, while in the New World the analogous attributes yielded pandemic strains, a self-limited epidemic strain (New Jersey), and semiendemic human-infecting strains, all porcine-derived H1N1 viruses.
- During spring and summer, subclinically infected migratory aquatic birds are apt to reach any frozen lake (and sea) across the subarctic and arctic upon thawing, and seed those lakes with viruses until freezing reoccurs (including in Greenland and the Arctic Ocean islands); during fall, parts of those bird populations are inclined to reach poultry farms, especially in China and Southeast Asia. Certain seals and whales apparently contribute to this seasonal dynamics across northern marine environments.
- The overall system of migratory aquatic bird pathways forms a global mosaic that interconnects in effect any water body worldwide, by means of migration routes of innumerable species; therefore, throughout any given whole year, any current or ice-released AIVs can potentially be conveyed between any two watery loci on Earth by means of one avian species, or, consecutively, by more than one species. The arctic tern (*Sterna paradisaea*), a species known to regularly migrate from pole to pole and host AIV [168], conspicuously illustrates this principle.

### 10.2. Principal Inferences at the Antigenic Level

- Any HA or NA antigenic subtype may potentially contribute to infectivity towards man, but very few HA-NA combinations (just 13 out of possible 144) proved infective in effect. Among those, H1, H2, H3, N1, N2, and N8 are distinctively predominant within pandemic strains; H5 and H7 (resembling the HPAI viruses) are relatively predominant within infective non-pandemic strains (thus far), while H9, H10, N3, and N7 comprise but singular episodes of human infection. Experimentally, H4 and H6 were found infective, while H11 was detected only serologically.
- Those different categories of antigenic subtypes appear to basically underlie the likelihood of their involvement in the generation of future pandemic strains, respectively. The level of synergism between the specifically combined HA and NA of a given strain is a major factor shaping its pandemic potential, for now exhibited in effect as H1N1, H2N2, H3N2, H2N8, and H3N8. All the other antigenic combinations isolated thus far from humans are less synergistic, representing aerogenically nontransmissible strains.
- Basically, there is consistent sequence in the recycling of the HA subtypes, namely, H1, followed by H2, followed by H3, followed by H1, and so on. This regularity has been prevailing for the last 163 years, at least, except for only the H2N8 1900 strain, which supposedly was, though, a singular case of an intra-human reassortant, as proposed above. The role of the NA subtype in that concern is apparently secondary.
- This antigenic sequence is sometimes interfered by the cocirculation of two subtypes, as were the cases of H3N8 together with H2N2 from 1889 to 1900, and H3N2 together with H1N1 from 1977 until present. Each of those two cases involved maximal antigenic subtypes (four), of which two were H3 and N2.
- Uniquely among mammals, all antigenic subtypes comprising pandemic strains are found within swine populations; whereas H1N1 is most predominant among pigs—and is regarded to be the porcine source of pandemic H1N1 strains—H3N2 is moderately common, but H2 and N8 are quite rare. Alongside, though, equine H3N8 strains were apparently involved in giving rise to the H3N8 and H3N2 pandemic strains, while the pandemic H3N8 virus plausibly reassorted with a preceding H2N2 strain, resultantly forming—but once, thus far, if at all—an intrahuman reassortant pandemic virus, namely, H2N8. Typically an equine antigenic subtype, H3N8 is but collaterally found within pigs [29] (and dogs [169]).
- A new pandemic strain of an antigenic subtype already circulating concomitantly appeared twice, involving, in both cases, the H1N1 subtype: in 1857, reportedly, and in 2009; such occurrence is not in conformity with the long accepted featuring of pandemic strains (in that a new antigenic subtype did not appear).
- In each of those two cases, an H1N1 strain first appeared in Asia (or Eurasia) (Table 3), followed by a distinctive H1N1 variant strain that appeared in Central America (Panama and Mexico). Further, the 2009 prototypic pandemic strain harbored two Eurasian genes, as was—according to at least one reference—also the case with part of the genome of the H1N1 1918 pandemic strain that surfaced in the USA.
- The genesis of at least two pandemic H1N1 strains (1918 and 2009) involved porcine genes and took place in swine (probably the 1918 strain and certainly the 2009 strain). Those two cases occurred in the Americas. Another swine-derived H1N1 strain, generating a focal epidemic among humans (New Jersey, 1976), singularly appeared too in the Americas.
- Bio-epidemiologically, contact transmission of non-aerogenic antigenic subtypes generates a temporary opportunity for airborne transmissibility (namely pandemic capacity) to form (through ongoing passages) within a given, new human-adapted strain, and if it does not form—which is assumingly usually the case—the new strain might just vanish (H1N1, New-Jersey, 1976; H7N7, The Netherlands, 2003), or persist but sporadically (H5N1, Southeast Asia, 2003–2011).

### 10.3. Principal Inferences at the Genomic Level

- Human and porcine influenza strains basically share the same gene pool, which is steadily enlarged by introduction of avian genes, mainly. Inputs of equine genes apparently took place, alongside.
- Predominantly, the viral human-avian genomic interface underlying the formation of pandemic strains is based upon surface antigen- and polymerase-encoding genes.
- Intact avian genotypes, although at times infective towards man and rarely thereafter transmissible (through contact, merely), are—perhaps inherently—not aerogenically transmissible, among humans, hence cannot generate pandemics.
- They might become pandemic subsequent to specific genomic alterations, chiefly through reassortment or recombination with mammalian strains. Sheer viral mutations taking place in a human being infected with an avian strain can doubtfully bring about airborne transmissibility (namely, pandemic capacity).
- Basically, human genotypes do not infect birds productively; on few occasions, they do infect turkeys in a proliferative manner.
- Genes of human (and porcine) strains are regularly introduced into the avian gene pool by transfections of reassortant viruses from swine to turkeys.
- In few cases, natural transfection from swine to wild ducks has been evidenced, albeit subclinical (triple, porcine-human-avian reassortants being involved), but the real extent of that potentially cardinal route of gene flow is actually unknown and might be rather wide.
- Due to the commonness of viral gene reassortments and the synergistic (as yet non-deciphered) versatility of gene constellation underlying infectivity and transmissibility, practically any viral gene (though not any genome), whether mammalian or avian derived, can potentially be contracted by migratory aquatic birds and thereby be disseminated and circulated worldwide within numerous waterborne strains.
- As a result, virtually any viral gene (though not any genome) can undergo preservation in annual or perennial environmental ice, thereafter reappearing genetically conserved to an extent that cannot be accounted for by ongoing regular mutational clock. This principle pertains as well to genes of human strains—in particular PB1, HA, and NA, at least—both pandemic and seasonal.
- Since genetic reassortments may expectedly occur instantly subsequent to ice thawing and consequent recontraction of ice-released viruses by aquatic birds, single gene segments demonstrating unordinary conservation are as representative of such preservation in ice as entire genomes (with which they were necessarily affiliated at some point in the past).

## Figures and Tables

**Table 1:** Evolutionary and anthropological events that ultimately gave rise to the emergence and proliferation of influenza type A strains within humans.

Event	Time (approximate)	Origin	Reference
Evolutionary emergence of first RNA viruses	3.5 billion years ago	Primeval biosphere	[6]
Evolutionary emergence of first wild ducks	65 million years ago	Archaic waterfowl	[7]
Evolutionary emergence of first influenza A virus	Unknown	Presumably wild duck intestine	[8]
Evolutionary emergence of first hominids	3.6 million years ago	Africa	[9]
Domestication of wild boar	8000 BC	China	[10]
Domestication of red junglefowl	8000 BC	Vietnam	[11]
Introduction of domestic junglefowl (chicken)	6000 BC	China	[11]
Domestication of wild duck (mallard)	5000 BC	China	[12]
Rise of first pandemic influenza strains	4000 BC	China (conceivably)	[13]
Domestication of wild horse	3500 BC	Kazakhstan	[14]
Domestication of wild turkey	1000 BC	Mexico	[15]
Domestication of wild quail	1100	China	[16]
Introduction of pigs into America	1539	Mexico Gulf coast	[17]

**Table 2:** Quantitative presence of avian and human influenza virus receptors in chicken, turkey, and duck.

	Chicken	Chicken	Turkey	Turkey	Duck	Duck
	AR*	HR**	AR	HR	AR	HR
Tracheal epithelium	80–90***	30–90	80–90	30–90	80–90	30–90
Intestinal epithelium	50–80	20–50	40–70	Negligible	40–70	Negligible

*Avian receptors; **Human receptors; ***values are given as percentage, expressing the extent of specific receptor occurrence, in relation to the total occurrence of sialic-acid-containing receptors.

**Table 3:** Referable influenza pandemics since 1510*.

Year	Geographic provenance	Antigenic subtype	Human genes	Avian genes	Porcine genes
1510^(A)^	Africa				
1557^(A)^	Asia				
1580^(A)^	Asia				
1732	Russia/USA				
1761^(A)^	Americas				
1781	China				
1800	Russia				
1830	China				
1847	Russia	H1N1^(A)^			
1857	Panama	H1N1(distinct variant)^(A)^			
1874^(B)^	Unknown	H2N2^(C)^			
1889	Kazakhstan^(D)^	H3N8^(E)^			
1900^(E)^	China^(F)^	H2N8^(E)^			
1918	USA	H1N1	PB2 NP NS	PA PB1	HA NA M
1957	China	H2N2	PA PB2 NP NS M	PB1 HA NA	None
1968	China	H3N2	PA PB2 NA NP NS M	PB1 HA	None
1977	China	H1N1	All genes	None	None
2009	Mexico	H1N1(distinct variant)	PB1	PA PB2	HA NA M NP NS

*Based on Beveridge [84], and modified according to additional sources as noted in the table and the following references: ^(A)^Taubenberger and Morens [85]; ^(B)^Tognotti [86]; ^(C)^Dowdle [87]; ^(D)^Hays [88]; ^(E)^Smith et al. [89]; ^(F)^Middle East Critical Care Assembly [90].

**Table 4:** Experimental infection of humans with LPAI strains.

Antigenic subtype	Strain	Infectivity (%)	Clinical reactions
H1N1	Dk/Alberta/35/76	20 (1/5)	None
H3N2	Dk/NY/6874/78	33 (1/3)	None
H3N8	Dk/Ukraine/1/63	50 (3/6)	None
H4N8	Dk/Alberta/288/78	23 (3/14)	Mild clinical symptoms
H6N1	Dk/Penn/486/69	18 (2/12)	Mild clinical symptoms
H6N2	Dk/Alberta/33/78	0 (0/5)	None
H9N2	Turkey/WI/1/66	18 (3/16)	None
H10N7	Turkey/MN/3/79	36 (6/16)	Mild clinical symptoms

**Table 5:** Antigenic subtypes of influenza A viruses that naturally infected man (based on various data presented above).

Antigenic subtype	Mode of occurrence	Place of occurrence
H1N1	Pandemic	Worldwide
H2N2	Pandemic	Worldwide
H3N2	Pandemic	Worldwide
H2N8	Pandemic	Worldwide
H3N8	Pandemic	Worldwide
H1N2	Epidemic (in 2001); sporadic	America, Asia, and Europe
H1N3	Singular	Azerbaijan
H5N1	Sporadically ongoing	Eastern Hemisphere
H7N2	Singular	USA
H7N3	Singular	Canada
H7N7	Limited epidemic	The Netherlands
H7N7 (further cases)	Singular	UK, USA (seal originated)
H9N2	Sporadic	China, Hong-Kong, and Bangladesh
H10N7	Singular	Egypt

**Table 6:** Genes contributed by avian genomes to pandemic influenza strains (based on various data presented above).

Pandemic strain	Avian genes contributed	Contemporary porcine input
1918—H1N1	PA PB1	Yes
1957—H2N2	PB1 HA NA	None
1968—H3N2	PB1 HA	None
2009—H1N1	PA PB2	Yes

**Table 7:** Contraction of pandemic/seasonal human influenza A virus genomes or genes by avian host species (based on various data presented above).

	Mode of contraction by			
Genome or genes contracted	Duck	Turkey	Quail	Chicken
Whole genome of H1N1 (different strains)	EI	UI and EI	EI	
Whole genome of H3N2 (different strains)	EI			EI
PB1, NA, and HA of H3N2	NI	NI	NI	
PB1 and NA of H1N2	NI	NI		

EI: experimental infection; NI: natural infection; UI: unintentional infection due to artificial insemination.
